# Enhanced Photocatalytic and Electrical Performance of Boron-Doped ZnO Nanorods: A Taguchi Optimization Approach for Degradation Efficiency

**DOI:** 10.1021/acsomega.5c11437

**Published:** 2026-05-25

**Authors:** Eray Tabak, Sadullah Öztürk, Arif Kösemen, Şahika Sena Bayazi̇t, Necmettin Kılınç, Mika Sillanpää, Birgül Benli̇

**Affiliations:** † 532719 Istanbul Technical University, Graduate School, Nano Science and Nano Engineering Programme, Istanbul 34469, Türkiye; ‡ Istanbul University-Cerrahpasa, Institute of Nanotechnology and Biotechnology, Istanbul 34500, Türkiye; § Health Biotechnology Joint Research and Applications Center of Excellence, Istanbul 34098, Türkiye; ∥ 37520 Inonu University, Faculty of Science & Arts, Department of Physics, Malatya 44280, Türkiye; ⊥ Saveetha School of Engineering, Saveetha Institute of Medical and Technical Sciences, Saveetha University, Chennai, Tamil Nadu 602105, India; # Institute for Nanotechnology and Water Sustainability (iNanoWS), Florida Campus, College of Science, Engineering and Technology, University of South Africa, Johannesburg 1709, South Africa; ∇ Centre of Research Impact and Outcome, Chitkara University Institute of Engineering and Technology, Chitkara University, Rajpura, Punjab 140401, India; ○ Istanbul Technical University, Faculty of Mines, Department of Mineral Processing Engineering, Istanbul 34469, Türkiye

## Abstract

In this study, the development of boron-doped zinc oxide nanorods (B-ZnO NRs) as an innovative photocatalyst for the degradation of organic pollutants under UV-A light was investigated. ZnO NRs, doped with varying concentrations of boron, were synthesized via a hydrothermal method, and their structural, optical, electrical, and photocatalytic properties were systematically characterized. The photocatalytic performance of B-ZnO NRs was evaluated under varying pH conditions, contaminant concentrations, and reaction times, while their electrical properties were analyzed by examining the conduction mechanism using the Arrhenius and Mott variable range hopping (VRH) conduction models. Results indicated that boron doping altered the conduction mechanism of ZnO as predicted by these models. In addition, boron doping enhanced electrical conductivity, with DC conductivity increasing up to 5-fold and alternating current conductivity by 3-fold compared to undoped ZnO. A significant enhancement in photocatalytic efficiency was observed, with B-ZnO exhibiting up to 140% higher degradation efficiency at pH 10 compared to pure ZnO. In addition, a Taguchi statistical optimization approach was employed to identify the most influential parameters affecting photocatalytic performance, including pH, reaction time, contaminant concentration, and boron doping levels. Optimal conditions were determined to be pH 10, a contaminant concentration of 2 μM, a reaction time of 90 min, and a boron doping level of 7%. Boron doping significantly improved the photocatalytic activity of ZnO nanorods, making them a promising candidate for advanced water treatment, hydrogen production, and environmental sensing applications.

## Graphical abstract

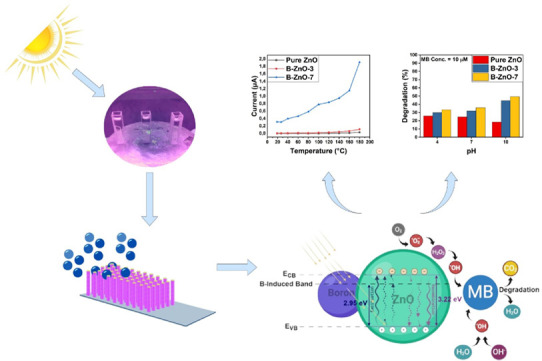

## 1 Introduction

Industrial development and technological advancements have led to rapid increases in environmental pollution, driven by growing populations, expanding agriculture, and industrial activity. The World Health Organization (WHO) estimates that environmental pollution is responsible for approximately 9 million deaths annually.^1^ Water pollution, which has become a major problem in recent times, is increased by many organic wastes along with increasing population and industrialization.^2^ These wastes contain harmful substances such as arsenic, nitrates, and chromium, which can cause severe health issues like cancer and other fatal diseases.^3,4^ Additionally, synthetic and organic dyes, primarily used in textiles, food, cosmetics, and medicine, contribute significantly to pollution. The global production of synthetic dyes alone reaches nearly 7 × 10^7^ tonnes per year.^5^ The textile industry releases large quantities of carcinogenic and toxic dyes into water bodies, rendering them unsuitable for consumption.^6^ Methylene blue, a dye commonly used in textile industries, is one of the most widely used organic dyes.^7^ In response to the rising pollution levels, research has focused on developing effective water treatment methods, including physical, chemical, and biological techniques like flotation, precipitation, solvent extraction, ion exchange, membrane filtration, biodegradation, and advanced oxidation processes (AOPs) such as photocatalysis.^8,9^

Photocatalysis is a promising technique that utilizes photocatalysts synthesized through simple synthesis methods, operates under light, and involves cost-effective systems. This process accelerates reactions by using light and photocatalysts, which absorb light to facilitate the reactions.^10^ For photocatalysis to occur, photocatalysts must form electron–hole (e^–^-h^+^) pairs capable of generating charge carriers when excited by light.^9,11^ This mechanism involves the reduction–oxidation reactions of excited electrons and oxygen radicals that efficiently degrade organic pollutants.^12–14^ Among various photocatalysts, metals, such as titanium dioxide (TiO_2_),^15^ ZnO,^16^ tungsten oxide (WO_3_),^17^ copper­(I) oxide (Cu_2_O),^18^ and cerium­(IV) oxide (CeO_2_),^19^ have demonstrated significant potential in pollutant degradation.^20,21^ In recent years, there has been an increase in the use of photocatalysts, leading to the development of new photocatalysts. Lin et al. have developed a new composite photocatalyst comprising tungsten nitride with nitrogen vacancies (W_2_N_3_–NV) and silver phosphate (Ag_3_PO_4_), which they have used to remove β-lactam antibiotics.^22^ They also synthesized a composite photocatalyst using Ag_3_PO_4_ and nitrogen-doped carbon (NC), achieving approximately 100% photocatalytic removal of norfloxacin, diclofenac, and phenol within 12 min.^23^ Lin et al. aimed to enhance H_2_O_2_ synthesis and optimize the electron transfer process using covalent organic framework (COF) structures. They demonstrated that COFs can significantly improve both proton conductivity and the efficiency of photoinduced charge separation by creating a hydrogen bond network through the immobilization of the H_3_PO_4_ molecular network onto COF nanochannels. This enhances photocatalytic performance in H_2_O_2_ production.^24^ Zhang et al. investigated the adsorption of heavy metals in wastewater using a vinyl-functionalized COF. Their composite photocatalyst simultaneously enabled the removal of tetracycline (TC) within 5 min.^25^ ZnO stands out as an ideal photocatalyst due to its large band gap energy (3.37 eV), high exciton binding energy (60 meV), biocompatibility, chemical stability, resistance to radiation, high photon absorption rate, and cost-effectiveness.^26–33^ However, ZnO’s photocatalytic efficiency is limited by challenges such as its wide band gap, rapid e^–^-h^+^ recombination, and photocorrosion under prolonged light exposure.^34^ To address these limitations, researchers have focused on doping ZnO with metals, nonmetals, or cosemiconductors.^35,36^ For instance, Zhang et al. enhanced ZnO’s surface area by six times through carbon doping, which reduced its band gap energy from 3.2 to 2.7 eV, enabling efficient visible light photocatalysis.^37^ Similarly, Poornaprakash et al. improved ZnO NRs’ photocatalytic properties through cobalt doping.^38^ Azfar et al. demonstrated the superior photocatalytic activity of Ag- and Ni-doped ZnO nanorods.^39^ Joy et al. showed that the band gap narrowed, and reactive oxygen species (ROS) production increased compared to pure ZnO with nonmetal doping (C, N, S), thereby proving an increase in photocatalytic efficiency.^40^ Sonkar et al. demonstrated that Cu doping reduced the band gap and increased the surface area.^41^ Meanwhile, Sakar et al. demonstrated the effects of metal doping, achieving this by narrowing the band gap and reducing recombination through Ce and Gd doping.^42^ Patil et al. synthesized Fe- and Cu-doped ZnO in a polythiophene matrix, demonstrating increased photocatalytic activity through band gap narrowing and heterojunction formation.^43^

Boron, a Group III element with high electronegativity and a small atomic radius, has shown great promise in improving ZnO’s photocatalytic performance.^44–47^ Boron doping enhances electron transfer by creating new energy levels within the band gap, which improves electron carrier concentration and reduces recombination.^48,49^ Recent studies, including those by Nguyen et al. have shown that boron-doped ZnO outperformed pure ZnO in photocatalytic applications,^50^ while Sharma et al. observed a 318% increase in photocurrent density for boron-doped ZnO nanorods.^51^ Similarly, Wang et al. reported that boron doping significantly enhanced the photocurrent density and photoelectric conversion efficiency of ZnO, indicating that boron doping reduced the recombination of photogenerated carriers.^52^ Atay and Gültepe reported that they successfully degraded methylene blue (MB) by 98% using 6% boron-doped ZnO films prepared via the sol–gel method.^53^ Nguyen et al. demonstrated that boron atoms act as electron traps in the band gap, thereby preventing recombination.^54^ In a subsequent study (2025), they proved that boron doping reduced the surface area and created surface defects.^55^ Putul et al. demonstrated through their analysis that superoxide (•O_2_^–^) and hydroxyl radicals (•OH) played a significant role in photodegradation.^56^ Furthermore, Nguyen et al. synthesized boron-doped ZnO/TiO_2_ and proposed that this increased the efficiency of photodegradation by reducing the recombination of e^–^-h^+^ pairs via the Z-scheme mechanism.^57^ The table below provides detailed information on studies conducted with boron doping and degradation rates. Upon careful examination of the table, the film structure is used very sparingly. However, in this study, the electrical conductivity properties were not investigated, and the photocatalytic mechanism related to the changes induced by boron doping was not discussed at the surface level. Additionally, the ZnO structure used was not in the form of nanorods but rather in thin film form.

This study distinguishes itself by combining boron doping with hydrothermal synthesis to fabricate ZnO nanorods exhibiting unique capped-sphere morphologies, which enhance surface area and charge carrier mobility. The novelty of this study lies in systematically demonstrating the critical role of boron doping in enhancing both the photocatalytic and electrical properties of ZnO NRs. Boron incorporation narrows the band gap, introduces new energy levels, and suppresses e^–^-h^+^ recombination, which collectively improve charge carrier density and photocatalytic efficiency. A detailed analysis of the electrical properties has revealed how boron doping alters the conduction mechanism of ZnO. Furthermore, by applying the Taguchi statistical optimization method, this work provides an original methodological contribution, identifying the most influential parameters and defining the optimum conditions for maximum degradation efficiency. Overall, the study highlights B-ZnO NRs as next-generation, sustainable photocatalysts with strong potential for environmental remediation and advanced semiconductor applications.

## 2 Experimental Section

### 2.1 Materials

Zinc acetate dihydrate (Zn­(CH_3_COO)_2_·2H_2_O), zinc nitrate hexahydrate (Zn­(NO_3_)_2_·6H_2_O), hexamethylenetetramine (C_6_H_12_N_4_), boric acid (HBO_3_), ethanol (absolute, ≥99.9%), acetone (≥ 99.9%), and MB were purchased from Sigma-Aldrich Co. and used without further purification. Microscope slides were obtained from Isolab Co. Only ultrapure water (18.2 MΩ·cm, Millipore) was used throughout the study. Isopropanol (IPA), l-ascorbic acid, and ethylenediaminetetraacetic acid (EDTA) were procured from Sigma-Aldrich.

### 2.2 Synthesis of ZnO Seed Layer

The preparation of pure ZnO NRs with hydrothermal method was previously published, and their gas-sensing properties were investigated.^58^ In this approach, ZnO NRs were grown on ZnO seed layer-coated glass substrates (1 cm × 2 cm) using the hydrothermal method. Microscope slides were first cut into controlled sizes (1 cm × 2 cm) using a diamond tip. They were then cleaned in acetone, ethanol, and ultrapure water using an ultrasonic bath for 10 min and subsequently dried. The growth of ZnO NRs was carried out in two steps: (i) deposition of a seed layer on the glass via the spin-coating method and (ii) hydrothermal growth of ZnO NRs.

For seed layer formation, 0.001 M zinc acetate dihydrate was dissolved in 50 mL of 99% pure ethanol. The solution was then stirred on a magnetic stirrer at 60 °C for approximately 40 min. A clean glass substrate was then placed in the spin-coating device, and 0.3 mL of the prepared solution was dropped onto its surface. The substrate was spun at 2500 rpm for 20 s and subsequently dried at 130 °C for 5 min. This process was repeated 5 times, after which the coated substrates were annealed at 300 °C for 1 h.

### 2.3 Growth of ZnO NRs and Boron Doping

For the hydrothermal growth solution, equimolar (0.01 M), Zn­(NO_3_)_2_·6H_2_O and C_6_H_12_N_4_ were dissolved in 80 mL of ultrapure water and stirred at room temperature for 1 h. After mixing, the solution was transferred to a glass bottle, and the glass substrates with the seed layer were immersed in the solution. The bottle was then tightly sealed and kept at 90 °C for 3 h to facilitate nanorod growth-controlled temperature and pressure conditions. After the reaction, the substrates were removed, rinsed with ultrapure water, and allowed to dry.

For boron doping, HBO_3_ was used as the boron source. Equimolar (0.01 M) zinc nitrate hexahydrate and hexamethylenetetramine were dissolved in 80 mL of ultrapure water, while boric acid was separately dissolved in ultrapure water under stirring. The dissolved boric acid was then added to the hydrothermal growth solution at concentrations of 3% and 7% by weight. The resulting samples were designated as B-ZnO-3 and B-ZnO-7, respectively. The same hydrothermal growth procedure was applied to obtain the B-ZnO NRs. As a result of these processes, three different samples were obtained. The synthesis steps are illustrated in Figure 1.

**Figure 1:**
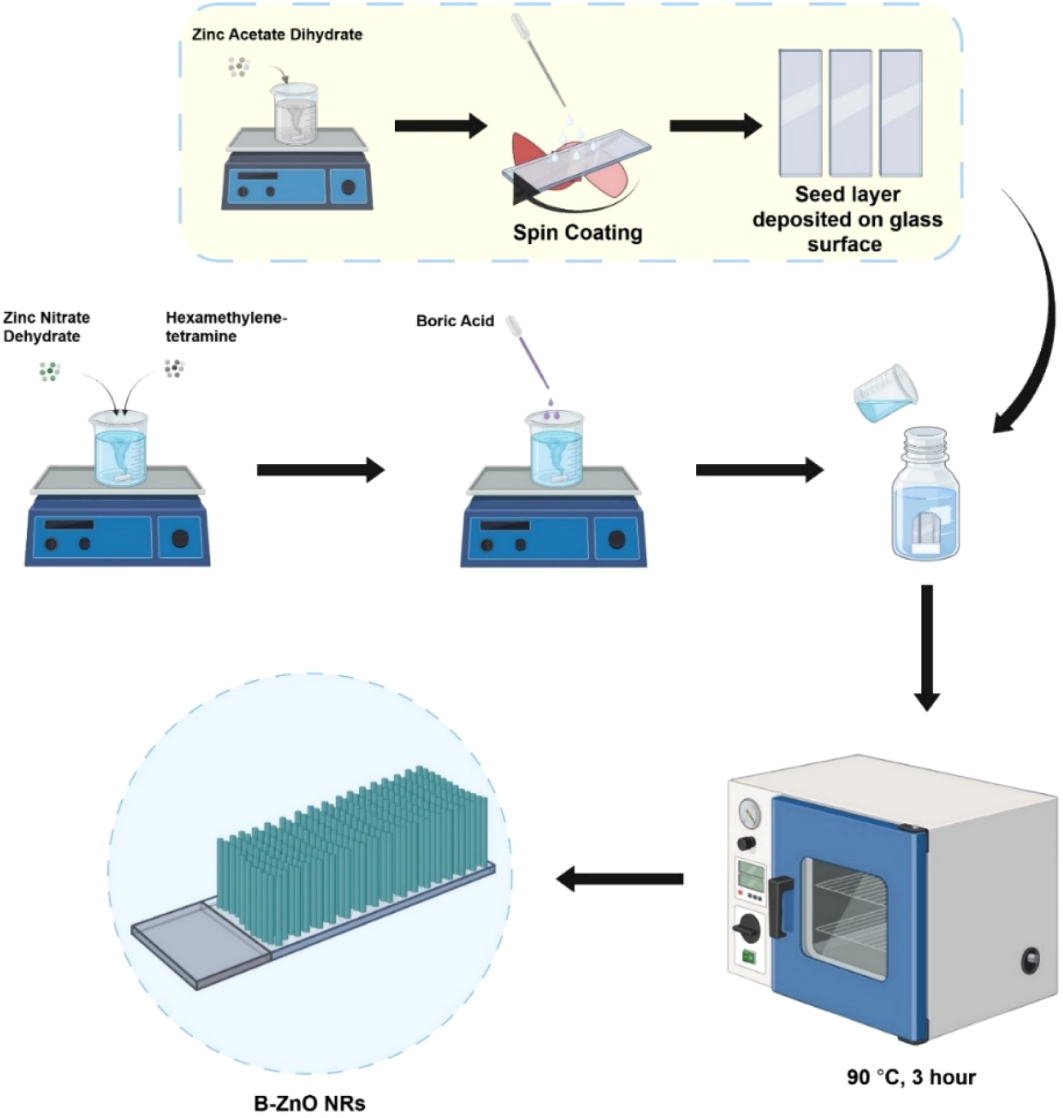
A schematic diagram for the synthesis of ZnO and B-ZnO NRs.

### 2.4 Characterization of ZnO Nanorods and B-ZnO NRs

X-ray diffraction (XRD) and Fourier Transform Infrared Spectroscopy (FT-IR) were used for structural analysis. XRD analysis (Rigaku, SmartLab) was performed to determine the crystal structures of the obtained samples by using CuKa1 radiation (λ = 0.15406 Å), with samples scanned in the 2θ range of 25–80° at room temperature.

The particle size was calculated using the Debye–Scherrer eq (eq 1), where the Bragg angle and the full width at half-maximum (fwhm) values were used:

1 $$D=\frac{K\lambda }{\beta_{hkl}\cos\theta }$$

Here, *D* represents the particle or crystal size, *K* is the Scherrer constant (approximately 0.9), λ is the wavelength of the incident X-ray (0.15406 nm), β is the fwhm in radians, and θ is the Bragg scattering angle. Crystal parameters were calculated using Bragg’s law (eq 2) and the interplanar spacing formula for a hexagonal system (eq 3):

2 $$n\lambda =2d_{hkl}\sin\theta$$

3 $$\frac{1}{d_{hkl}^{2}}=\frac{4}{3}\left(\frac{h^{2}+hk+k^{2}}{a^{2}}\right)+\frac{l^{2}}{c^{2}}$$

Here, *n* is the diffraction order (typically *n* = 1), *d_hkl_* is the interplanar spacing for a given Miller index (*h*, *k*, *l*), and *a* and *c* are the lattice parameters of the hexagonal system.

FT-IR analysis was conducted using a Jasco FT/IR-4700 spectrometer at room temperature in the wavenumber range of 500–4000 cm^–1^. The optical properties and absorption characteristics were examined using UV–vis spectrometry (Thermo Scientific: Multiskan Go) in the wavelength range of 200–800 nm. The band gap energy was calculated from the absorption peaks using the Tauc method (eq 4):

4 $$\alpha h\nu =A{\left(h\nu -E_{g}\right)}^{n}$$

where α is the absorption coefficient, hυ represents the photon energy, *E_g_* is the band gap, *A* is a proportionality constant, and *n* denotes the transition type. Since ZnO exhibits a direct band gap transition, *n* was taken as 2. X-ray Photoelectron Spectroscopy (XPS, Thermo K-Alpha X-ray Photoelectron Spectrometer) and Wavelength Dispersive X-ray Fluorescence (WD-XRF, Rigaku Primus II) were used to determine the surface chemical composition, elemental distribution, and oxidation states of the elements in the sample.

Surface morphology and topographical features were analyzed using a scanning electron microscope (SEM, ZEISS Evo LS 10). Additionally, atomic force microscopy (AFM, XE-70E; Park Systems Corp., Suwon, Korea) was employed to examine the surface topography of ZnO NRs. Measurements were conducted in contact mode using NSC36/Cr–Au-type cantilevers at a 0.5 Hz scanning speed under moisture-controlled conditions (22 ± 2 °C). Prior to each experiment, cantilevers were UV/ozone-treated (UV Cleaner, Bioforce Nanosciences) for 15 min to remove contaminants. The nanorod dimensions and surface features were analyzed using XEI Image Processor (Park Systems Corp., Suwon, Korea).

The point of zero charge (pH_pzc_) is the pH at which the surface charge of the material in solution is zero.^59^ The pH_pzc_ values of ZnO and B-ZnO photocatalysts were determined using the pH drift method.^60^ In this method, a 0.1 M NaCl solution was prepared and adjusted to pH values of 4, 7, and 10 using HCl and NaOH. The photocatalysts were added to these solutions and stirred at room temperature for 48 h. The final pH values were measured, and the pH_pzc_ was determined by calculating the difference between the initial pH and the final pH values.

### 2.5 Electrical Analysis

The electrical properties of the obtained samples, pure ZnO NRs, B-ZnO-3 NRs, and B-ZnO-7 NRs, along with the temperature-dependent variations in DC and AC currents, were investigated and analyzed. Before measurements, samples were coated by using a thermal evaporation method (NANOVAK PVD system) with silver (Ag) to obtain two contact electrodes that have 0.2 mm space and 2 × 5 mm area. Measurements were conducted in a dry, air-controlled environment, with a continuous flow of 200 standard cubic centimeters per minute (sccm) of high-purity nitrogen gas through the measurement cell.

For DC characterization, a voltage was applied to the samples incrementally, starting from 0 V and increasing up to 2 V in 0.05 V steps at specific time intervals. Once 2 V was reached, the voltage was symmetrically decreased to −2 V and then brought back to 0 V. This cycle was repeated twice, and the corresponding voltage and current variations were recorded under each temperature condition.

### 2.6 Photodegradation Tests

Photocatalytic tests were conducted in a closed environment under UV-A light (CAMAG, 8W, λ = 365 nm). MB was used as the organic contaminant and prepared at two different concentrations, 2 μM and 10 μM. The pH was adjusted using diluted HCl and NaOH, and experiments were carried out at three different pH levels: 4, 7, and 10. Each prepared solution (3 mL) was transferred to precleaned quartz cuvettes. To evaluate the photocatalytic activity, B-doped and undoped ZnO-coated glass substrates were immersed in the MB solution. The samples were initially kept in the dark for 40 min to establish adsorption–desorption equilibrium. Subsequently, they were exposed to UV-A light for 90 min to induce photodegradation. The degradation process was monitored using a UV–vis spectrometer by tracking the absorbance changes over time. The concentration of methylene blue (MB) was quantified using a calibration curve derived from the absorbance–concentration relationship, as shown in (eq 5):

5 $$C=\frac{A-b}{m}$$

where *C* represents the MB concentration, *A* is the corresponding initial absorbance value, *b* is the intercept at the calibration curve, and *m* is the slope at calibration curve. The degradation efficiency (%) was calculated using (eq 6):

6 $$\mathrm{Degradation rate}\left(\%\right)=100\left(1-\frac{C_{t}}{C_{0}}\right)$$

where *C*_0_ is the initial MB concentration, and *C*_*t*_ is the concentration at time *t*.

To analyze the reaction kinetics, both the pseudo-first order and pseudo-second-order kinetic models were applied. The pseudo-first-order kinetics were determined using the logarithmic relationship (eq 7):

7 $$\ln\left(\frac{C_{t}}{C_{0}}\right)=-k_{1}t$$

where *k*_1_ is the rate constant (min^–1^). For the pseudo-second-order kinetics, the rate equation was given below (eq 8):

8 $$\frac{1}{C_{t}}-\frac{1}{C_{0}}=k_{2}t$$

where *k*_2_ represents pseudo-second-order rate constant.

### 2.7 Taguchi Approach for Degradation Experiments

The Taguchi method is a robust statistical technique widely used for optimizing and improving experimental parameters.^61^ It employs a special orthogonal array design, allowing the evaluation of multiple process parameters with a minimal number of experiments. The method relies on the signal-to-noise (S/N) ratio to quantify the deviation of experimental factors from desired values. The S/N ratio shows the relationship between the useful outcome (signal) and the variability of measured values (noise).^62^ A higher S/N ratio corresponds to more optimal results, thereby simplifying the optimization process.^63^ In this study, the Taguchi method was applied using Minitab Statistical Software to analyze experimental results by converting them into S/N ratios. The three common Taguchi optimization criteria are 1. Nominal is better (used when the target value is a specific level), 2. Larger is better (applied when higher values yield superior performance), 3. Lower is better (used when lower values indicate improved performance). For this study, four factors were selected, and a mixed-level (2–3) Taguchi design was chosen. The selected factors and levels are presented in Table 1. The “larger is better “criterion was employed for optimization, and the corresponding S/N ratio was calculated using eq 9:

$$\frac{S}{N}=-10\;\log\left(\frac{1}{n}\overset{n}{\underset{i=1}{\sum }}\frac{1}{y_{i}^{2}}\right)$$ 9

where *y* represents the performance characteristic value of the *i* experiment, *i* denote the experimental trial number , and *n* the total number of experimental observations is the total number of experimental observations is the number of experiments.

**Table 1:** Factors and Levels of Taguchi Design

Factors/Levels	pH	MB Concentration (μM)	Time (min)	Doped (wt %)
1. Level	4	2	30	0
2. Level	7	10	60	3
3. Level	10		90	7

In accordance with the selected factors and their respective levels, the L18 orthogonal array was employed. The chosen factors include pH, reaction time, initial concentration, and doping amount, with the degradation rate as the response variable. Since a higher degradation rate is preferred, “larger is better” criterion was applied. The experimental design is summarized in Table 2.

**Table 2:** Experimental Conditions for the Taguchi Design

pH	Time (min)	MB Concentration (μM)	Doped (wt %)
4	30	2	0
7	60	2	0
10	90	2	0
4	30	2	3
7	60	2	3
10	90	2	3
7	30	2	7
10	60	2	7
4	90	2	7
10	30	10	0
4	60	10	0
7	90	10	0
7	30	10	3
10	60	10	3
4	90	10	3
10	30	10	7
4	60	10	7
7	90	10	7

## 3 Results and Discussions

### 3.1 Structural Properties

Figure 2a presents the XRD patterns of pure ZnO, B-ZnO-3, and B-ZnO-7 NRs synthesized via the hydrothermal method. All samples exhibited the characteristic diffraction peaks of the hexagonal wurtzite ZnO phase (JPCDS No. 36–1451), with no additional peaks associated with secondary phases or impurities, confirming phase purity and successful boron incorporation. The dominant (002) peak observed at ∼34.67° demonstrated strong preferential growth along the *c*-axis, typical of vertically aligned nanorod morphologies. Such uniaxial orientation minimized surface energy and represented a hallmark of anisotropic ZnO crystal growth under hydrothermal conditions.

**Figure 2:**
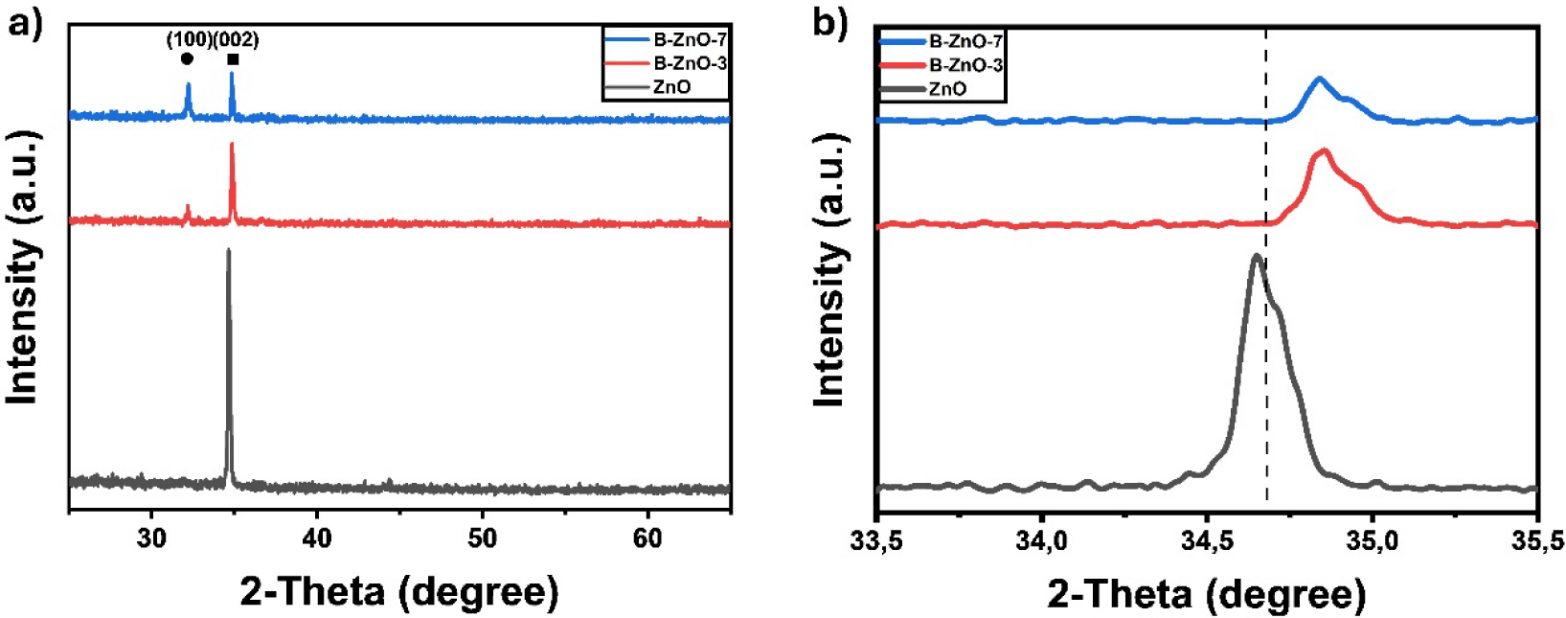
a) XRD patterns of pure and B-ZnO NRs. b) Magnified view of the (002) peak at 2θ = 34.67° in doped and undoped NRs.

Boron doping in ZnO could occur through three possible mechanisms.^49^ In ZnO, oxygen ions (O^2–^) formed a tightly packed hexagonal lattice, while zinc ions (Zn^2+^) occupied tetrahedral or octahedral vacancies. The octahedral vacancies, consisting of six triangular faces and two parallel bases rotated by 60°, were typically unoccupied. In n-type ZnO, with higher Fermi levels, these vacancies were more likely to be filled. Alternatively, boron ions (B^3+^) could replace either Zn^2+^ or O^2–^ within the lattice. Wang et al. reported that B^3+^ incorporation into octahedral vacancies might cause peak shifts in XRD patterns.^52^ Such shifts served as evidence of structural defects arising from boron doping in the hexagonal wurtzite crystal structure. Since B^3+^ (0.23 Å) had a significantly smaller ionic radius than Zn^2+^ (0.74 Å), substituting Zn or O atoms with boron led to lattice contraction. This, in turn, resulted in peak shifts toward higher angles, in accordance with Bragg’s law.^64^

The observed shift in the (002) peak at 2θ = 34.67° (Figure 2b) suggested successful boron doping, consistent with previous reports.^65^ Interestingly, the (002) peak intensity of B-ZnO-7 is slightly lower than that of B-ZnO-3, which may have indicated a partial relaxation of lattice strain at higher B concentrations. Increased dopant content could lead to defect reorganization or the formation of less oriented domains, thereby reducing peak intensity despite further peak shifting. Table 3 presented the crystallite size, calculated using the Scherrer equation and lattice parameter. As expected, boron doping generally reduced crystal size due to structural distortion. A slight decrease was observed at 3% doping, while the 7% doped sample exhibited a marginal increase, possibly due to local lattice relaxation. The narrowing of lattice parameters supported the substitution of Zn^2+^ and O^2–^ atoms by B^3+^.

**Table 3:** Structural Parameters of Pure and B-ZnO NRs

Samples	2θ_(0 0 2)_ (°)	fwhm_(0 0 2)_ (°)	C (Å)	D (nm)
Pure ZnO	34.67	0.17322	5.1699	48.05
B-ZnO-3	34.87	0.18318	5.1416	45.46
B-ZnO-7	34.88	0.17258	5.1396	48.25

The emergence of the (100) peak at ∼31.7° in boron-doped samples suggested a mild loss of preferential orientation and the presence of polycrystalline domains. However, no foreign phase peaks were detected, and the (100) reflection is consistent with ZnO’s wurtzite structure. This indicated that boron incorporation altered crystallographic texture rather than inducing secondary phases. The (100) reflection was likely associated with orientation randomness or increased disorder arising from dopant–lattice mismatch. The full JCPDS reference data set used for peak indexing was provided in the (Supporting Information Table S1).

FT-IR analysis was used to further confirm the successful incorporation of boron into the ZnO structure. The FT-IR spectra of both doped and undoped ZnO NRs, recorded in the range of 500–4000 cm^–1^, were presented in Figure 3. The peaks observed between 400 and 600 cm^–1^ corresponding Zn–O stretching^66^ in the hexagonal wurtzite structure.^67^ Boron incorporation introduced additional peaks in the 1300–1700 cm^–1^ range, which were attributed to B–O stretching and B–O–B bending vibrations.^68^ According to Molla et al., peaks in the 1600–700 cm^–1^ region were indicative of B–O vibrations, confirming the presence of boron within the ZnO matrix.^69^ As shown in Figure 3, the intensity of the peaks due to B–O stretching absorption between 1300 and 1500 cm^–1^ increased with increasing dopant concentrations, further supporting the successful doping of ZnO NRs with boron.^48,68^

**Figure 3:**
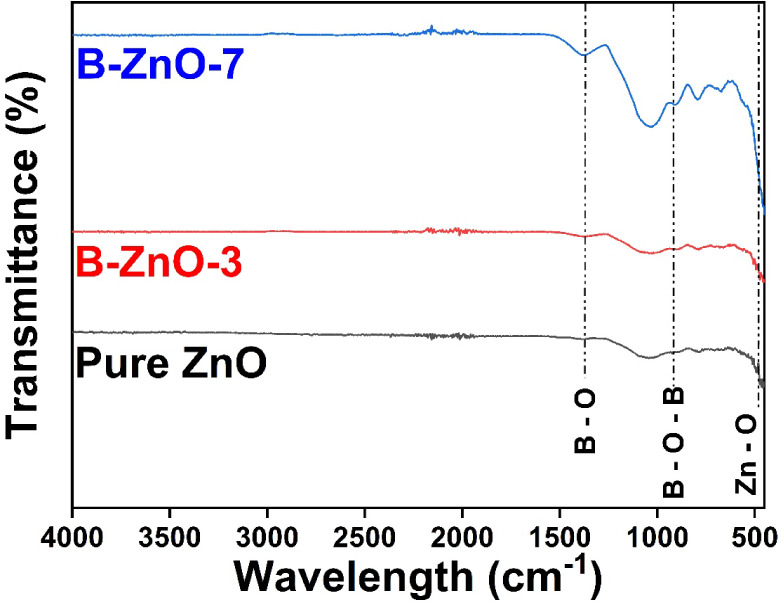
FT-IR spectra of pure and B-ZnO NRs.

In ZnO nanostructures doped with boron, the B 1s XPS signal was generally very weak and often could not be detected at all. This situation has been frequently reported in the literature, especially in structures with nanorod morphology and low doping concentrations.^70–72^ No direct B 1s signal was obtained in XPS analysis; however, this did not imply that boron doping did not occur.

The effect of boron doping on the oxygen/zinc (O/Zn) atomic ratio in ZnO thin films was quantitatively analyzed using XPS. According to Table 4, the O/Zn ratio in pure ZnO was determined to be 1.33, which likely indicates an oxygen-rich surface due to adsorbed species or small structural defects. Following B-ZnO-3, the O/Zn ratio increased significantly to 2.05. This increase indicated a notable enrichment of surface oxygen species, consistent with boron’s role in promoting the formation of oxygen vacancies and chemically adsorbed oxygen or hydroxyl groups. In the corresponding O 1s spectrum, a significant increase was observed in the oxygen peak associated with defects (∼531.5 eV), confirming the structural effect of boron doping on the ZnO matrix. At B-ZnO-7, the O/Zn ratio increased further to 2.24, indicating continued oxygen accumulation on the surface. However, this increase rate was lower compared to the transition from ZnO to B-ZnO-3, which suggested a saturation trend in oxygen uptake or defect formation at higher doping levels. This situation demonstrated the limits of boron’s structural or chemical incorporation into the ZnO lattice before phase separation or secondary effects begin. In general, the gradual increase in the O/Zn ratio with increasing boron doping clearly demonstrated that boron doping effectively modulated the surface chemistry and defect structure of ZnO. This enhances the material’s potential for applications such as gas sensors, catalysts, or other surface-interactive applications. The saturation trend observed at higher doping levels might indicate the optimal doping threshold for maximizing surface reactivity, thereby demonstrating the limits of doping without compromising material stability.

**Table 4:** XPS Spectroscopy Analysis of the Zn 2p and O 1s Areas, and the O/Zn Ratio

Sample	O 1s Area	Zn 2p Area	O/Zn Ratio	Defect O (%)
ZnO	39454	130111	1.33	66.1%
B-ZnO-3	52154	110868	2.05	70.8%
B-ZnO-7	46528	90810	2.24	87.9%

The significant increase in the O/Zn ratio in the doped samples and the shifts in binding energy observed in the Zn 2p and O 1s peaks in Figure 4 indicate that the structure has an oxygen-rich and degraded environmental structure. Similarly, in the study by Pal et al.,^71^ the B 1s signal was not observed by XPS; however, the electrical, optical, and structural characterizations of the structure supported the presence of boron doping. Similarly, in systems such as C_3_N_4_/ZnO, it has been reported that the Fermi level shifts approximately 0.4 eV upward with boron doping.^73^

**Figure 4:**
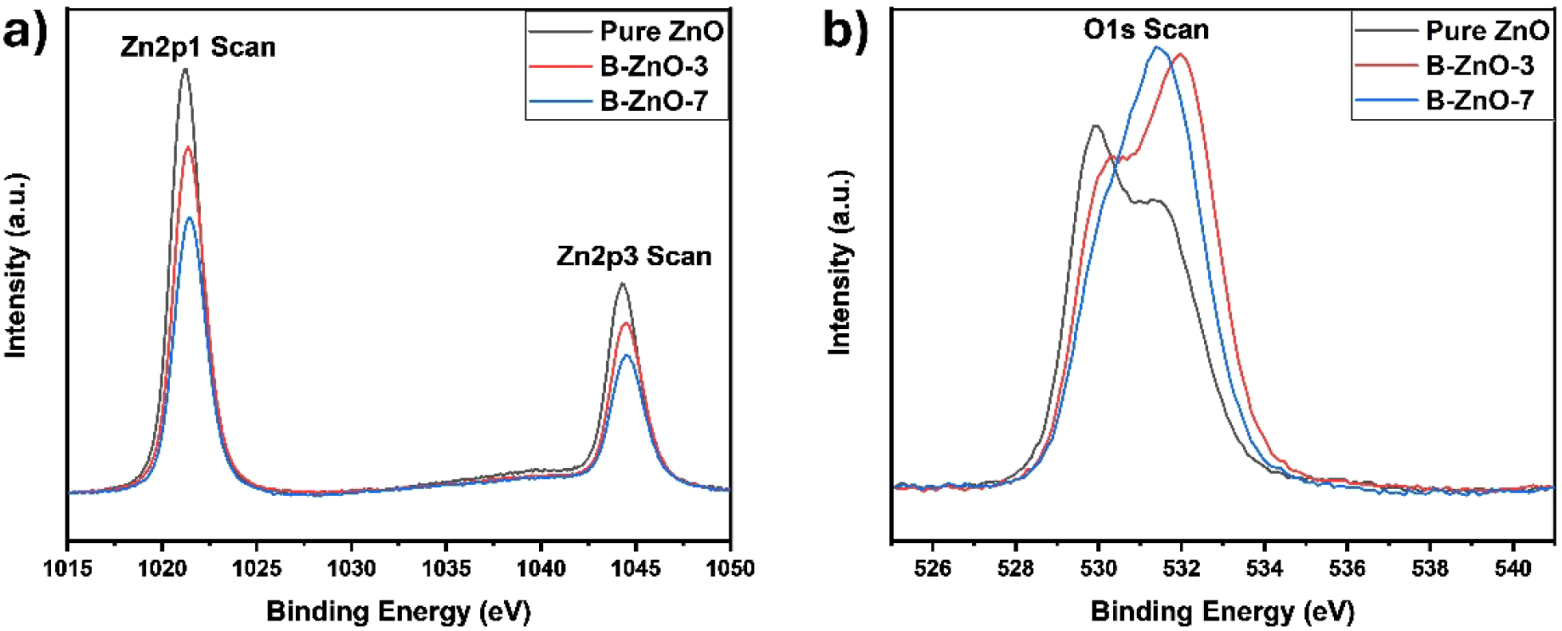
a) Zn 2p1 and Zn 2p3 scan. b) O 1s scan of all samples.

Although no distinct B 1s peak was observed in the XPS spectra, this does not necessarily indicate the absence of boron incorporation. In boron-doped ZnO nanostructures, particularly those with nanorod morphology and low doping concentrations, the B 1s signal is frequently reported to be extremely weak or undetectable due to its low surface atomic concentration and small photoionization cross-section.^70–72^ Therefore, indirect yet widely accepted indicators were considered to evaluate boron incorporation. The systematic increase in the O/Zn atomic ratio (Table 4), the significant enhancement of the defect-related O 1s component (∼531.5 eV), and the observable binding energy shifts in the Zn 2p and O 1s spectra (Figure 4) collectively confirm modification of the ZnO lattice and surface chemistry upon boron addition. These combined structural and electronic changes provide strong evidence for successful boron incorporation, consistent with previous reports in the literature. WD-XRF analysis was performed to verify the boron content. As can be seen in Figure S7 and Table S2, the boron content was successfully determined to be close to the specified values.

### 3.2 Morphological Properties

The surface morphology of the synthesized samples was characterized using SEM and AFM, and the corresponding images are shown in Figure 5. In Figure 5a, hexagonal nanorod growth was successfully achieved for pure ZnO despite the presence of vacancies on the surface. AFM images (Figure 5b) further supported this observation, revealing a morphology consistent with the hexagonal wurtzite crystal structure. In contrast, the SEM image of B-ZnO-3 (Figure 5c) indicated the formation of surface defects due to boron doping. As the boron concentration increases, these defects become more pronounced, leading to spheroidization in the structure, as observed in Figure 5d.^65^ Additional SEM images (Figures S1–S3) demonstrated that a reduction in nanorod diameter results in an increase in surface area. These findings aligned with the particle sizes given in Table 3, which were derived from XRD analysis.

**Figure 5:**
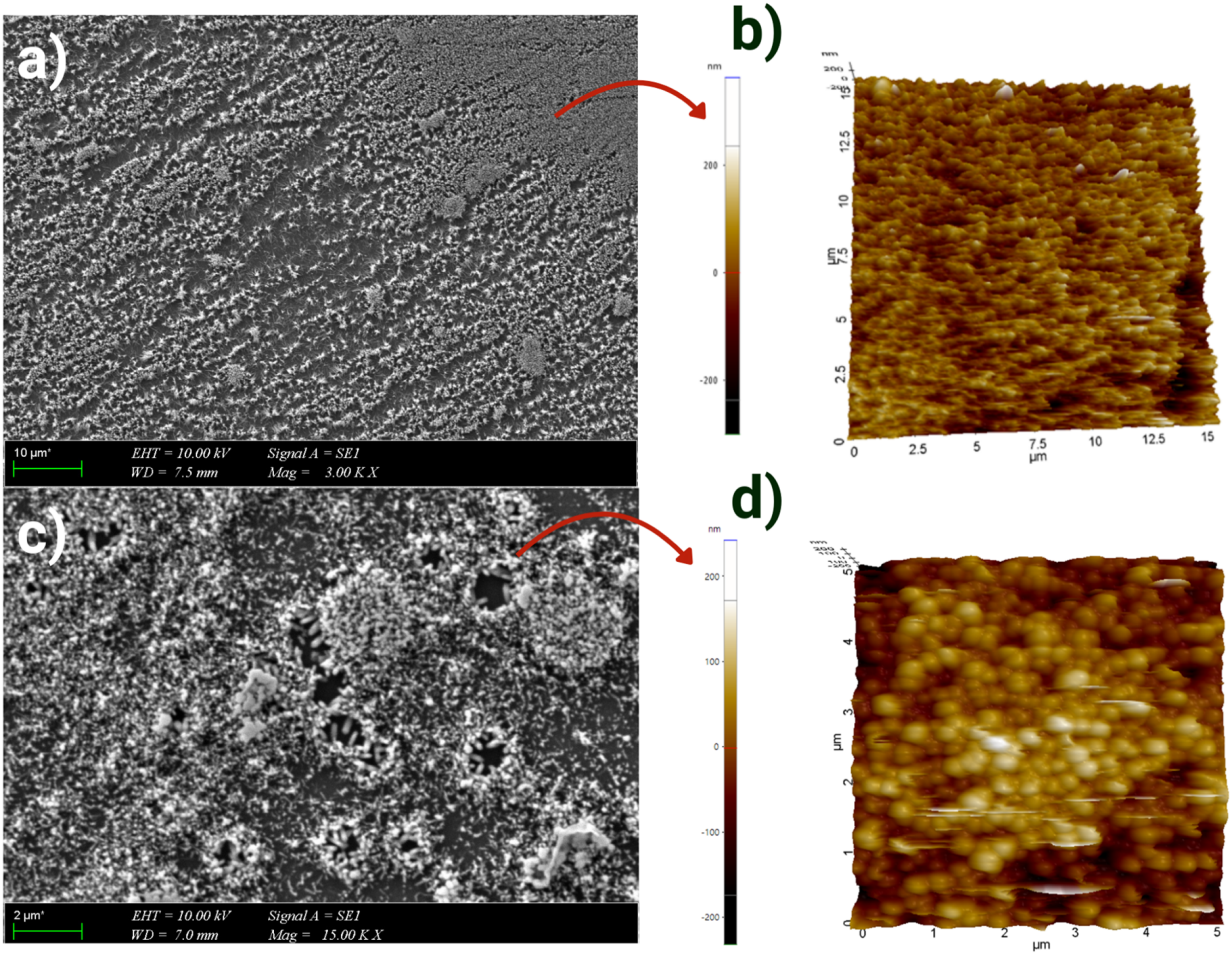
SEM and AFM images: a) SEM image for pure ZnO, b) AFM images for pure ZnO, c) SEM image of B-ZnO-3, d) AFM image of B-ZnO-3.

The XRD patterns further indicated that increasing the boron doping concentration led to the substitution of Zn^2+^ in the ZnO lattice with B^3+^. This substitution altered the local electric field generated by dipole interactions, thereby restricting the nanorod growth. The enhanced intensity of the (100) peak observed in the XRD patterns at higher doping levels can be attributed to the modified electric field interactions. Moreover, SEM images (Figures S1–S3) confirmed that boron doping limits the elongation of nanorods, supporting the findings from both XRD and AFM analyses. These results demonstrated that boron doping significantly influenced the crystal size, surface morphology, and structural properties of ZnO. Thus, the doping concentration played a crucial role in controlling the nanostructure properties of ZnO, offering valuable insights for future applications.

### 3.3 Optical Absorption of B-ZnO NRs

The optical properties of the synthesized pure and B-ZnO NRs were analyzed using UV–vis spectrophotometry in the 200–800 nm wavelength range (Figure 6a). The absorption peaks for ZnO, B-ZnO-3, and B-ZnO-7 occurred at 352, 361, and 363 nm, respectively. These results indicated a red shift of the absorption peak toward the visible region due to boron doping, with a clear increase in the wavelength of the absorption peak. The shift of the absorption peaks correlated well with changes in the band gap energies of the samples. The band gap energies for pure ZnO, B-ZnO-3, and B-ZnO-7 were calculated to be 3.22, 3.02, and 2.95 eV, respectively. These values are depicted in Figure 6b. According to Salas et al., boron doping in ZnO caused a shift of the absorption peak toward the visible region and led to a decrease in the band gap energy, resulting from revealing new energy levels as electrons occupied the conduction band in doped ZnO.^68^ Similarly, Furka et al. reported that doping created adjacent bands, reducing the energy required for electron transitions between the valence and conduction bands.^74^ This reduction in transition energy was reflected in the decrease in the band gap energy and the red shift of the absorption peaks observed in this study. Among the samples, B-ZnO-7 exhibited the lowest band gap energy, indicating that boron doping effectively narrows the band gap energies compared with pure ZnO.

**Figure 6:**
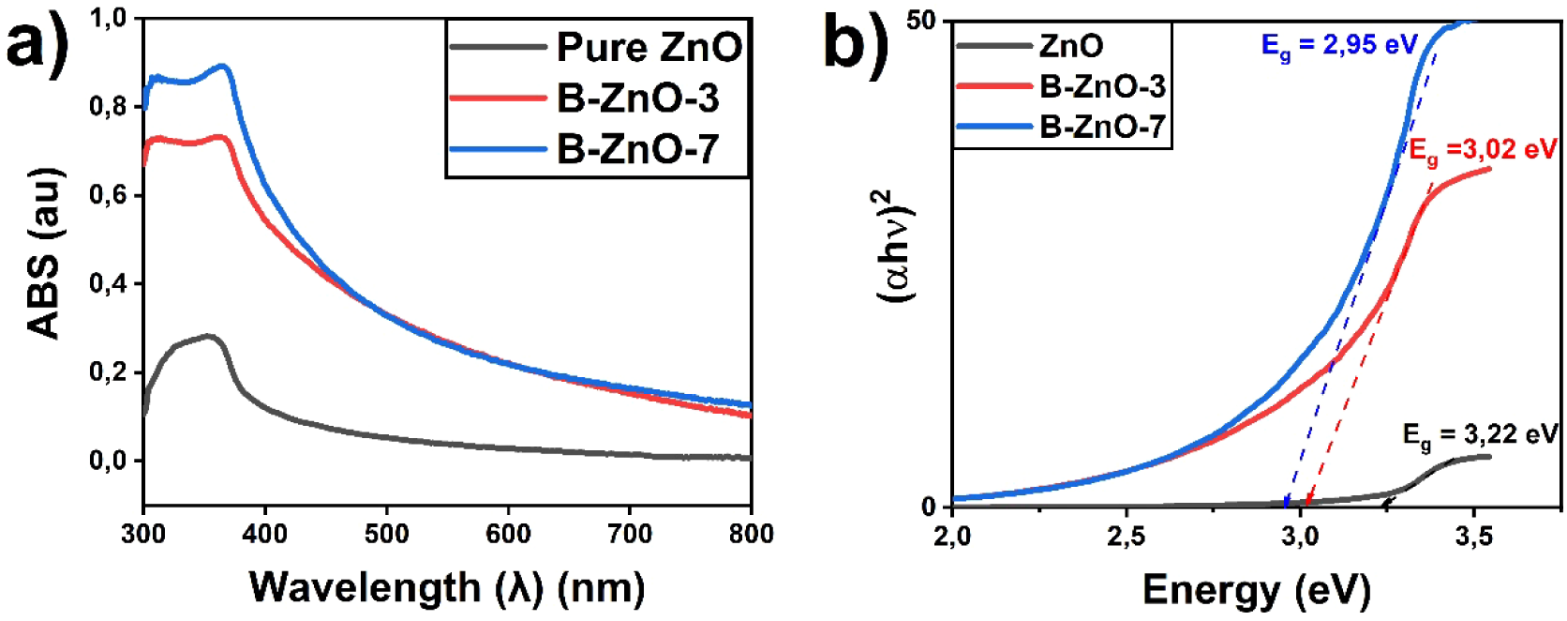
a) UV–vis absorption spectra of all samples. b) Band gap energies calculated using the Tauc method.

### 3.4 Electrical Properties

Figure 7 shows the current at an applied voltage of 2 V and its variations with temperature for pure ZnO, B-ZnO-3, and B-ZnO-7 samples. ZnO is known to be an n-type semiconductor material.^75^ As depicted in Figure 7a, pure ZnO exhibited a sharp increase in current with increasing temperature, which was characteristic of typical semiconductor behavior.^58^ Figure 7b and c present the current-temperature curves for B-ZnO-3 and B-ZnO-7, respectively. While pure ZnO (Figure 7a) showed exponential behavior, the current response for the B-doped samples transitioned from exponential to linear as the doping level increased. This change in behavior suggested that boron doping altered the conduction mechanism in ZnO. During the formation of ZnO, boron doping compensated for the electrons removed from the crystal structure by the oxygen atoms, incorporating them back into the ZnO lattice. This doping effect enhanced the electrical conductivity, depending on the doping concentration. Therefore, boron doping modified the electrical conductivity by adding more electrons to the ZnO crystal structure.

**Figure 7:**
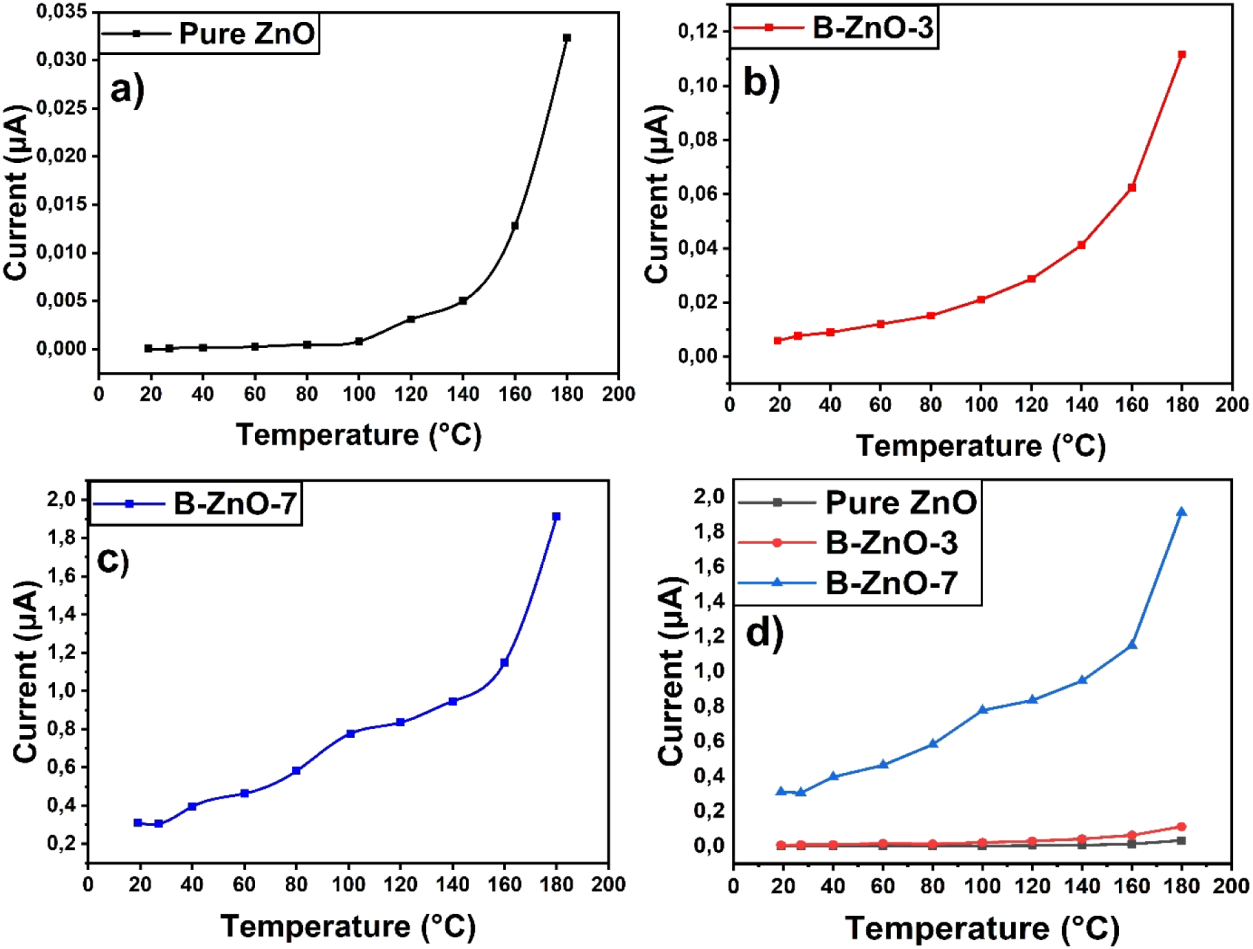
a) Temperature-dependent DC current for pure ZnO, b) for B-ZnO-3, and c) for B-ZnO-7. d) Comparison of temperature- dependent DC current for pure ZnO and boron-doped ZnO samples.

Figure S4 presented the I–V characteristics for pure ZnO, B-ZnO-3, and B-ZnO-7 samples. All three graphs showed a gradual increase in current with increasing voltage. For boron-doped samples, the current increased with the doping level, with B-ZnO-7 exhibiting a roughly 5-fold increase in current compared to pure ZnO. Figure 8 showed the change in AC conductivity in low frequency (500 Hz) of pure ZnO, B-ZnO-3, and B-ZnO-7 samples as a function of temperature. As previously mentioned, ZnO is an n-type semiconductor, and the exponential increase in current observed for pure ZnO was expected. As seen in Figure 8, the conductivity of ZnO increased with boron doping in the ZnO crystal lattice. The conductivity values for pure ZnO, B-ZnO-3, and B-ZnO-7 were 2.37 × 10^–8^ S, 5.98 × 10^–8^ S, and 6.76 × 10^–7^ S, respectively. Compared to pure ZnO, B-ZnO-7 exhibited an increase of approximately 300%. This suggested that boron doping significantly enhanced the electrical properties of ZnO, leading to improved electron donation and transport, which could consequently increase photocatalytic efficiency.

**Figure 8:**
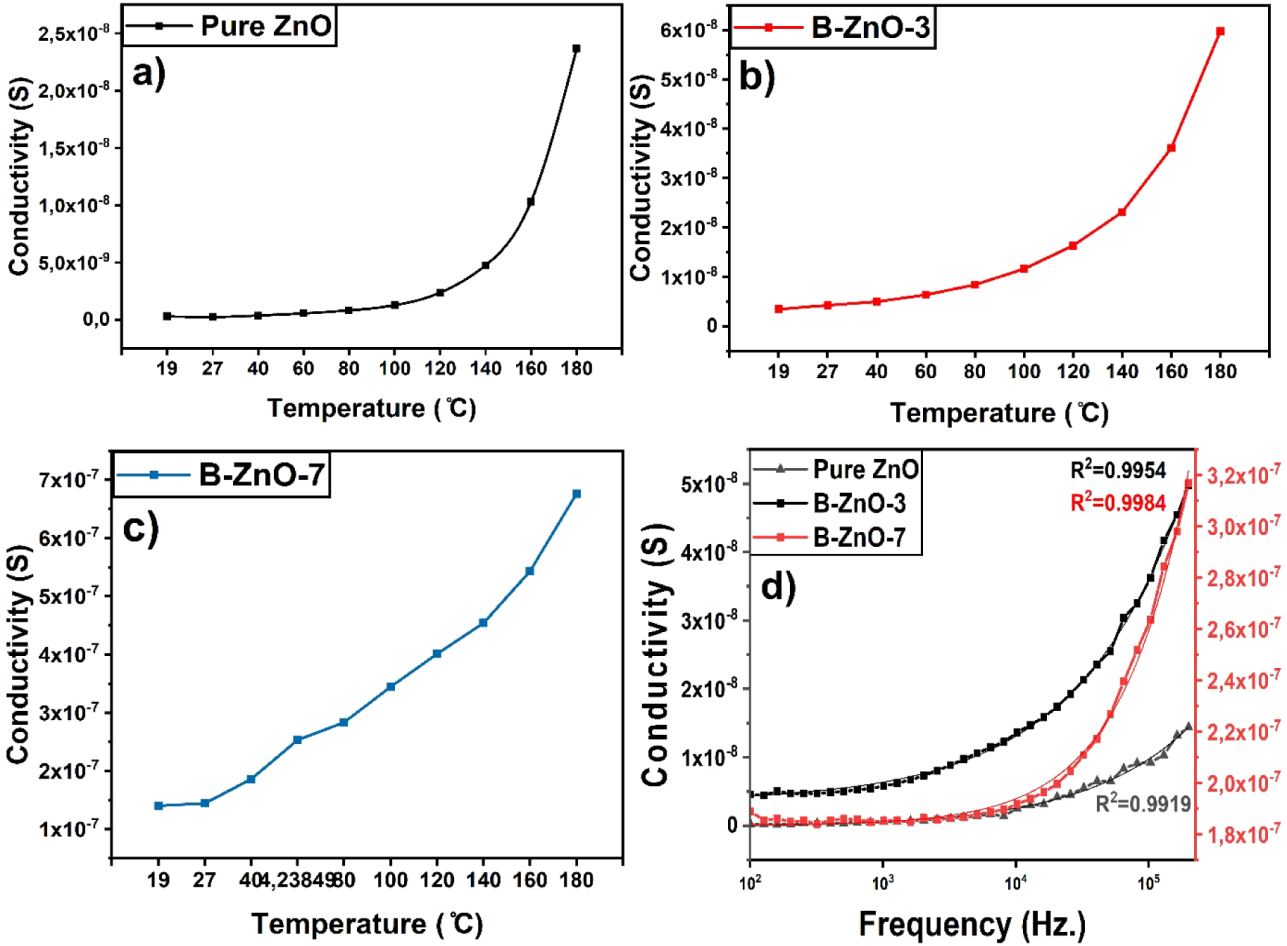
a) Temperature-dependent AC current for pure ZnO, b) for B-ZnO-3, and c) for B-ZnO-7. d) Comparison of frequency—dependent AC current for pure ZnO and boron-doped ZnO samples at 40 °C.

Figure 9 shows the electrical mechanisms as a function of temperature, which were analyzed using the Arrhenius model (Figure 9a) and the VRH model (Figure 9b). In this study, DC electrical conductivity values derived from Johnscher Power Law (eq 10) were analyzed with both Arrhenius and Mott VRH models:

10 $$\sigma_{ac}=\sigma_{dc}\left(T\right)+A\omega^{n}$$

where σ_*ac*_ is the AC conductivity, σ_*dc*_ is the DC conductivity, and Aω^n^ is a frequency-dependent conductivity term. The temperature dependence of the conductivity was calculated according to the Arrhenius Law (eq 11):

11 $$\sigma_{dc}=\sigma_{0}\exp\left[-\frac{E_{a}}{k_{B}T}\right]$$

where *E*_a_ is the activation energy, *T* is the temperature, *k*_*B*_ is the Boltzmann constant, and σ_0_ is a constant of proportionality.

**Figure 9:**
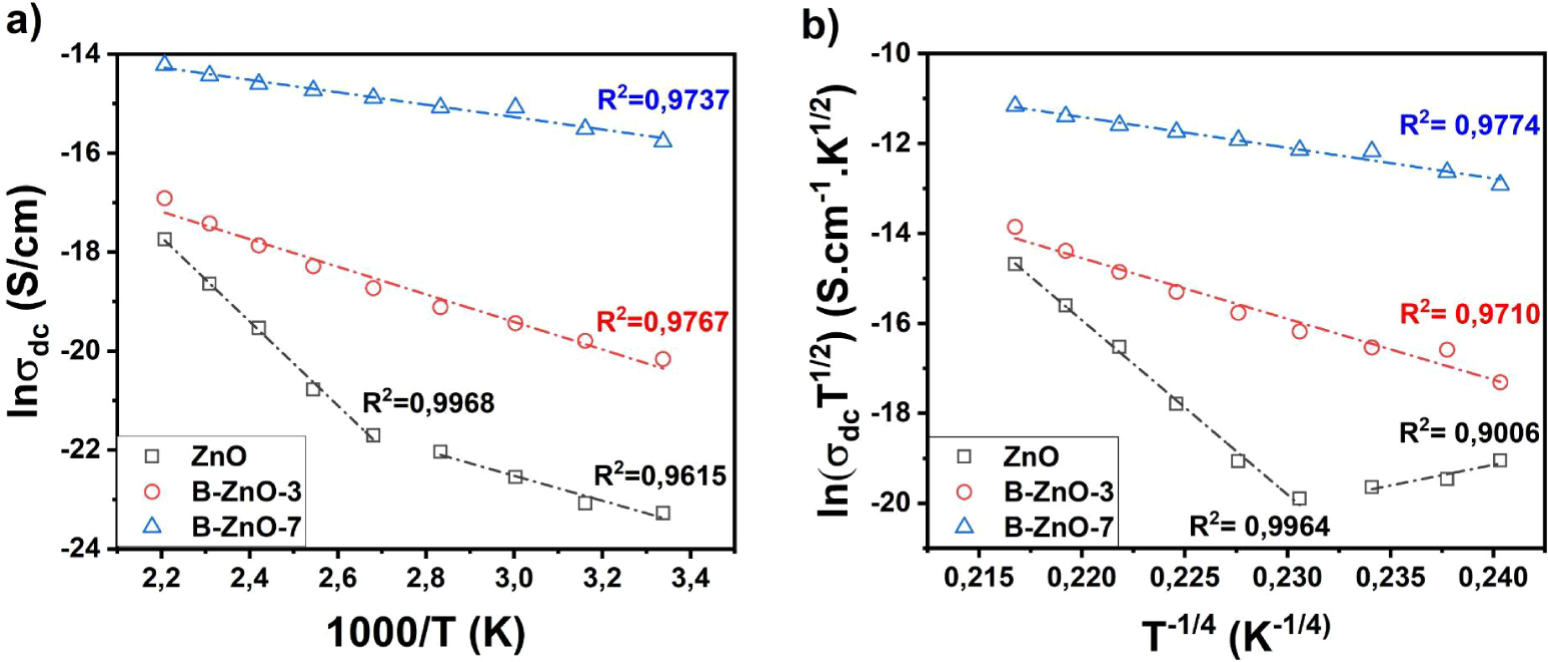
a) Plots of lnσ versus 1000/T­(K) of ZnO, B-ZnO-3, B-ZnO-7, and b) plots of ln­(σ.*T* ^1/2^) versus *T* ^–1/4^ is, B-ZnO-3, B-ZnO-7 NRs measured to 300–453 K.

In the high-temperature region, the temperature-dependent DC conductivity was modeled using the Mott VRH (eq 12):

12 $$\sigma_{dc}T^{1/2}=\sigma_{0}\exp\left[-{\left(\frac{T_{1}}{T}\right)}^{1/4}\right]$$

From Figure 9a, the electrical conductivity increased with boron doping. The activation energies calculated for ZnO, B-ZnO-3, and B-ZnO-7 were 0.732, 0.24, and 0.108 eV, respectively.^76^ According to this model, ZnO showed low conductivity with an R^2^ value of 0.9968, demonstrating lower compatibility in the low-temperature range but higher compatibility at higher temperatures. The decrease in activation energy with increasing boron doping indicated that more electrons were entering the conduction band with temperature, resulting in enhanced conductivity.^77^ According to Figure 9b, the linear relationship between ln­(σ.*T* ^1/2^) and *T* ^–1/4^ confirmed that carriers move between localized energy levels via quantum tunneling at low temperatures. For ZnO, the R^2^ value at low temperatures was 0.90, a less optimal fit of the VRH model in this regime. In contrast, B-ZnO-3 and B-ZnO-7 exhibited R^2^ values of 0.9710 and 0.9774, respectively, indicating a better fit the VRH model and higher conductivity at low temperatures. Consequently, in the high-temperature regime (Arrhenius model), electrons increased the conductivity by transferring to the conduction band via thermal activation. Boron doping enhanced conductivity by facilitating this thermal activation process, while in the low-temperature regime (VRH model), the quantum tunneling mechanism dominated, and boron doping improved carrier mobility by lowering energy barriers.

### 3.5 Photocatalytic Activity of B-ZnO and Mechanisms of Photodegradation

B-doped and undoped ZnO samples (2 cm^2^, one-side coated) were vertically immersed in MB solutions with varying pH levels (4, 7, 10) and concentrations (2, 10, 50 μM). Negligible degradation was observed when MB was exposed to irradiation without a catalyst, confirming the necessity of photocatalysis. The adsorption–desorption equilibrium occurring in the dark region is shown in Figure S5. pH plays a crucial role in photocatalytic reactions in aqueous solutions by influencing both the photocatalyst’s behavior and the pollutant’s properties. The degradation mechanisms involve •OH attack, direct oxidation by positive holes, and reduction by conduction band electrons, all of which contribute to dye decomposition.^78^ In this study, pH values of 4, 7, and 10 were selected to systematically evaluate photocatalytic performance under different chemical conditions, representing acidic, neutral, and basic environments, respectively. These pH ranges are reference values commonly used in photocatalytic degradation studies.

To ensure adsorption–desorption equilibrium, the samples were first in the dark for 60 min before being exposed to UV-A radiation for 90 min. The degradation rates after 90 min are presented in Figure 10. The results indicated that the degradation efficiency decreased with increasing MB concentration. For each pH and concentration level, B-ZnO samples showed significantly higher degradation than undoped ZnO.

**Figure 10:**
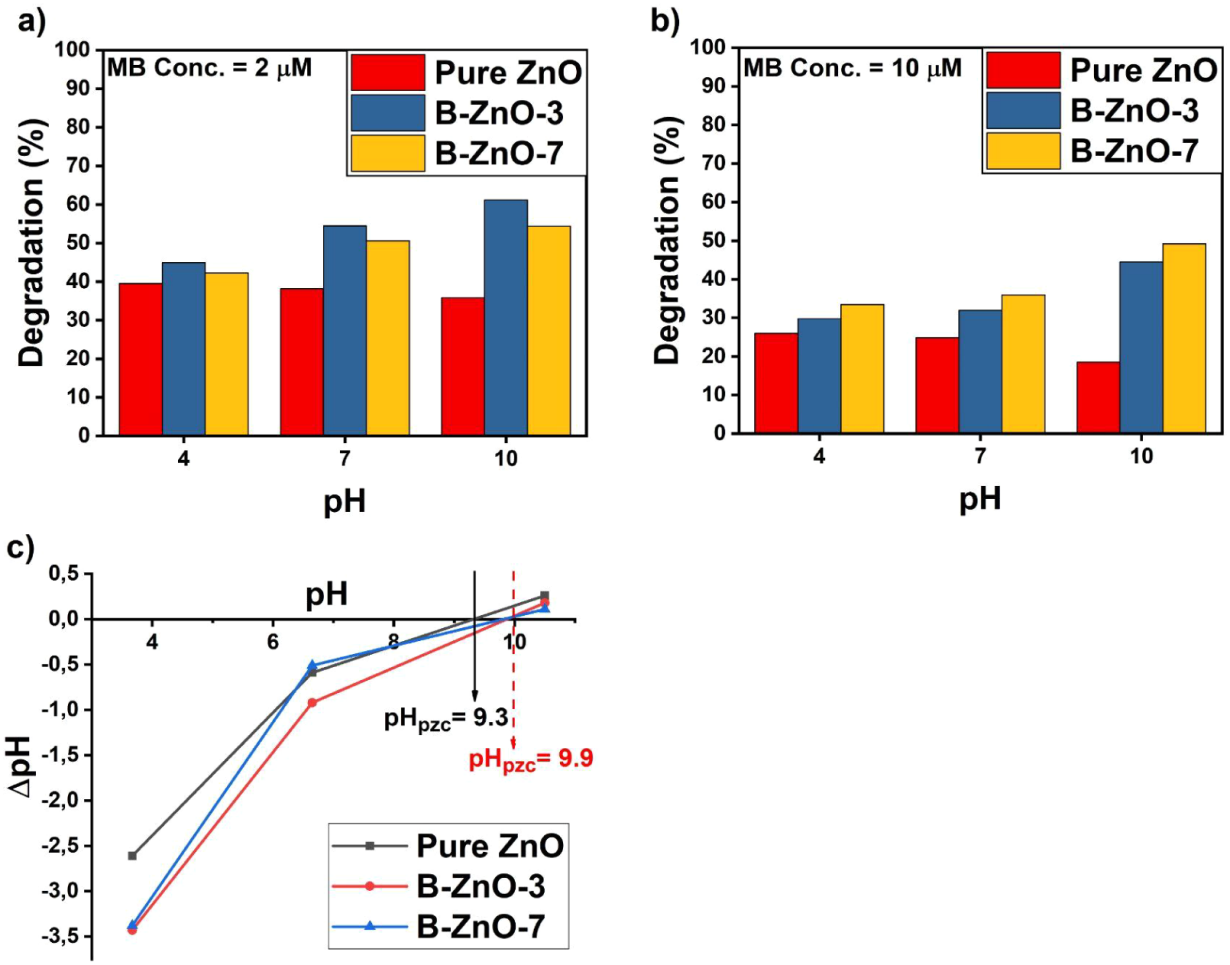
At the end of 90 min, the degradation rate (%) of all samples: a) pH-dependent degradation rates at 2 μM concentration, b) pH-dependent degradation rates at 10 μM concentration, and c) pH_pzc_ plot of ZnO and B-ZnO photocatalysts.

As shown in Figure 10a, at a 2 μM MB concentration, B-ZnO-3 demonstrated a 13% higher degradation than pure ZnO at pH 4, a 42% higher degradation at pH 7, and a 70% higher degradation at pH 10. Similarly, a 10 μM concentration (Figure 10b), B-ZnO-7 exhibited a 14% higher degradation than pure ZnO at pH 4, a 28% higher degradation at pH 7, and a 140% higher degradation at pH 10. These results indicated that that MB photodegradation decreased with increasing pH for pure ZnO, whereas it increased with increasing pH for B-ZnO. Additionally, Figure S6 illustrates the change in MB concentration as determined by UV–vis spectroscopy. The absence of new peaks in the MB spectrum throughout the reaction also indicates that no new chromophoric groups are formed.^7^

Figure 10c presented the point of zero charge (pH_pzc_) values for ZnO and B-ZnO samples, showing neutrality at pH 9.3 for ZnO and pH 9.9 for B-ZnO. Since MB is a cationic dye at any pH value,^79^ the electrostatic interactions between the catalyst surface and MB play a crucial role in adsorption. At pH < pH_pzc_ (e.g., pH 4), ZnO surfaces are positively charged, reducing MB adsorption due to electrostatic repulsion. However, in acidic environments, •OH formation is limited, making hole (h^+^) oxidation the dominant degradation mechanism. The presence of dissolved oxygen in acidic conditions, also enhanced •O_2_^–^ formation, which can further contribute to MB photodegradation.^80^ According to Mohabansi et al., ZnO showed better photodegradation in acidic media due to surface effects and interactions with ionizable organic molecules.^81^ In contrast, B-ZnO exhibited enhanced photodegradation efficiency at higher pH levels, primarily due to stronger electrostatic attraction between the negatively charged photocatalyst surface and the positively charged MB dye.^82^ Additionally, higher pH levels promoted hydroxyl ion (OH^–^) availability, leading to increased •OH formation and improved degradation efficiency. •OH’s accelerated photodegradation by breaking MB’s conjugated structure, ultimately leading to its complete mineralization into CO_2_ and H_2_O.^83^

To understand the role of radicals in photocatalytic degradation, radical scavenger tests were conducted under constant conditions. Ascorbic acid, isopropanol (IPA), and ethylenediaminetetraacetic acid (EDTA) were used to investigate the roles of •O_2_^–^, •OH, and photogenerated h^+^, respectively.^82,84–86^ A 1 mM scavenger was added to a 2 μM MB solution and tested with B-ZnO-7. As shown in Figure 11a, the B-ZnO-7 photocatalyst exhibited a 72% removal rate in the absence of a scavenger. The MB removal rates were 43%, 10%, and 5% with the addition of IPA, EDTA, and ascorbic acid, respectively. Inhibiting active radicals with various scavengers significantly reduced the degradation of the MB dye. While all radicals contribute to the degradation of the MB dye, h^+^ and •O_2_^–^ radicals play a particularly significant role in its photocatalytic degradation under UV-A light. The reusability of the B–ZnO-7 photocatalyst was investigated under constant conditions (initial MB concentration: 2 μM; pH: 7; irradiation time: 90 min) for up to five cycles, and the results are shown in Figure 11b. The photocatalyst was washed with water and ethanol, dried, and then reused. No significant decrease in removal efficiency was observed with repeated use of the photocatalyst. The material’s removal efficiency remained consistent over five cycles. This further demonstrates the stability of the B–ZnO NR structures grown on the surface. Therefore, it can be concluded that B–ZnO is suitable for the long-term and repeated removal of dyes from textile wastewater.

**Figure 11:**
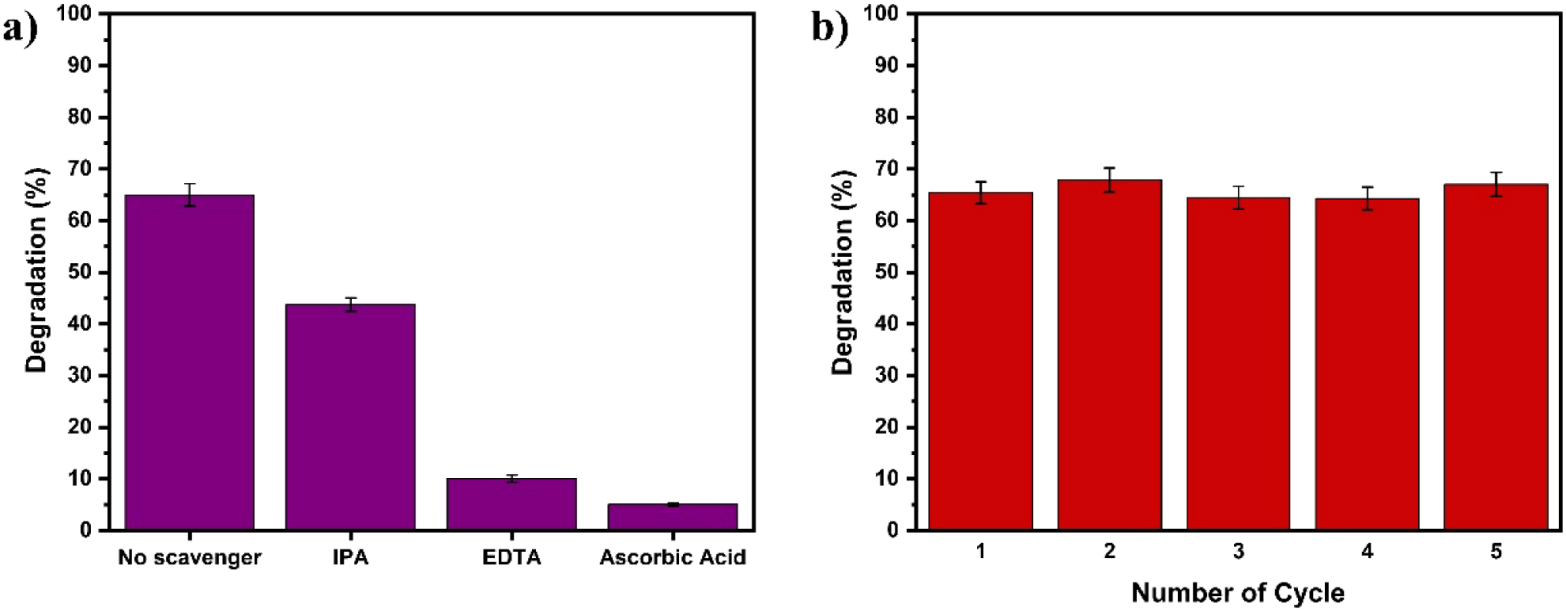
a) Role of radical scavengers in the photocatalytic removal of MB with B–ZnO-7 (initial MB concentration: 2 μM; concentration of all scavengers: 1 mmol/L; pH: 7; irradiation time: 90 min) and b) reusability of B-ZnO-7 (initial MB concentration: 2 μM; pH: 7; irradiation time: 90 min).

Figure 12 presents the time-dependent ln­(C/C_0_) values calculated based on the pseudo-first-order kinetic model under different pH and concentration conditions after 90 min of irradiation. Both pseudo-first- and pseudo-second-order kinetic models were evaluated to determine the adsorption mechanism of ZnO and B-ZnO NRs. The rate constants and R^2^ values obtained from the pseudo-first-order kinetic model are given in Table 5.

**Figure 12:**
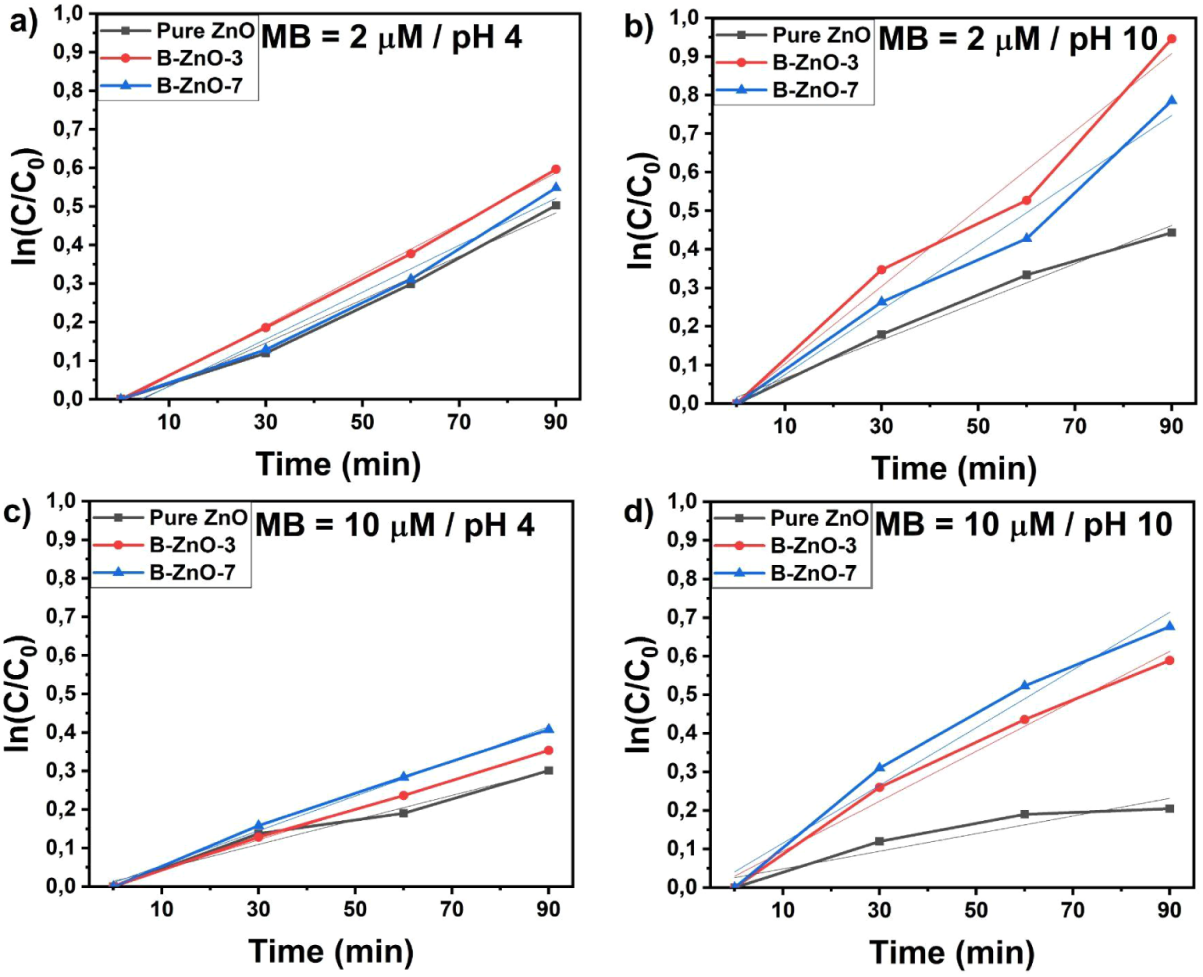
Graphs of pseudo-first kinetic models: a) time-dependent ln­(C_0_/C) graphs for 2 μM MB, pH 4, b) time-dependent ln­(C_0_/C) graphs for 2 μM MB, pH 10, c) time-dependent ln­(C_0_/C) graphs for 10 μM MB, pH 4 , and d) time-dependent ln­(C_0_/C) graphs for 10 μM MB, pH 10.

**Table 5:** Pseudo-First-Order Kinetics of Pure and B-ZnO NRs

Sample Name	MB Concentration (μM)	pH	Kinetic Rates (1/min^–1^)	R^2^
ZnO	2	4	0.005624	0.98723
		7	0.005484	0.97073
		10	0.004949	0.98905
B-ZnO-3	2	4	0.006606	0.99848
		7	0.008766	0.99716
		10	0.010063	0.97949
B-ZnO-7	2	4	0.006093	0.98272
		7	0.007915	0.99616
		10	0.008402	0.98001
ZnO	10	4	0.003185	0.9743
		7	0.003111	0.99607
		10	0.002282	0.8943
B-ZnO-3	10	4	0.003895	0.9989
		7	0.004261	0.99881
		10	0.006474	0.9840
B-ZnO-7	10	4	0.004495	0.9962
		7	0.00489	0.99419
		10	0.007476	0.9760

As shown in Figure 12, the MB concentrations decreased with increasing irradiation time. The highest degradation rate constants (0.01006 min^–1^) were observed for B-ZnO-3 NRs at pH 10 with a 2 μM MB concentration, while B-ZnO-7 exhibited the highest rate constant (0.00666 min^–1^) at pH 10 with a 10 μM concentration. These results indicated that B-ZnO NRs consistently exhibited higher rate constants than undoped ZnO for all pH and concentration conditions, further confirming the efficiency of boron doping in enhancing photodegradation.

Figure 13 and Table 6 provide additional data on pseudo-second-order kinetics, with R^2^ values indicating that both ZnO and B-ZnO predominantly follow the first-order kinetic model. This suggested that photodegradation was primarily governed by physical adsorption and electrostatic interactions on the photocatalyst surface.

**Figure 13:**
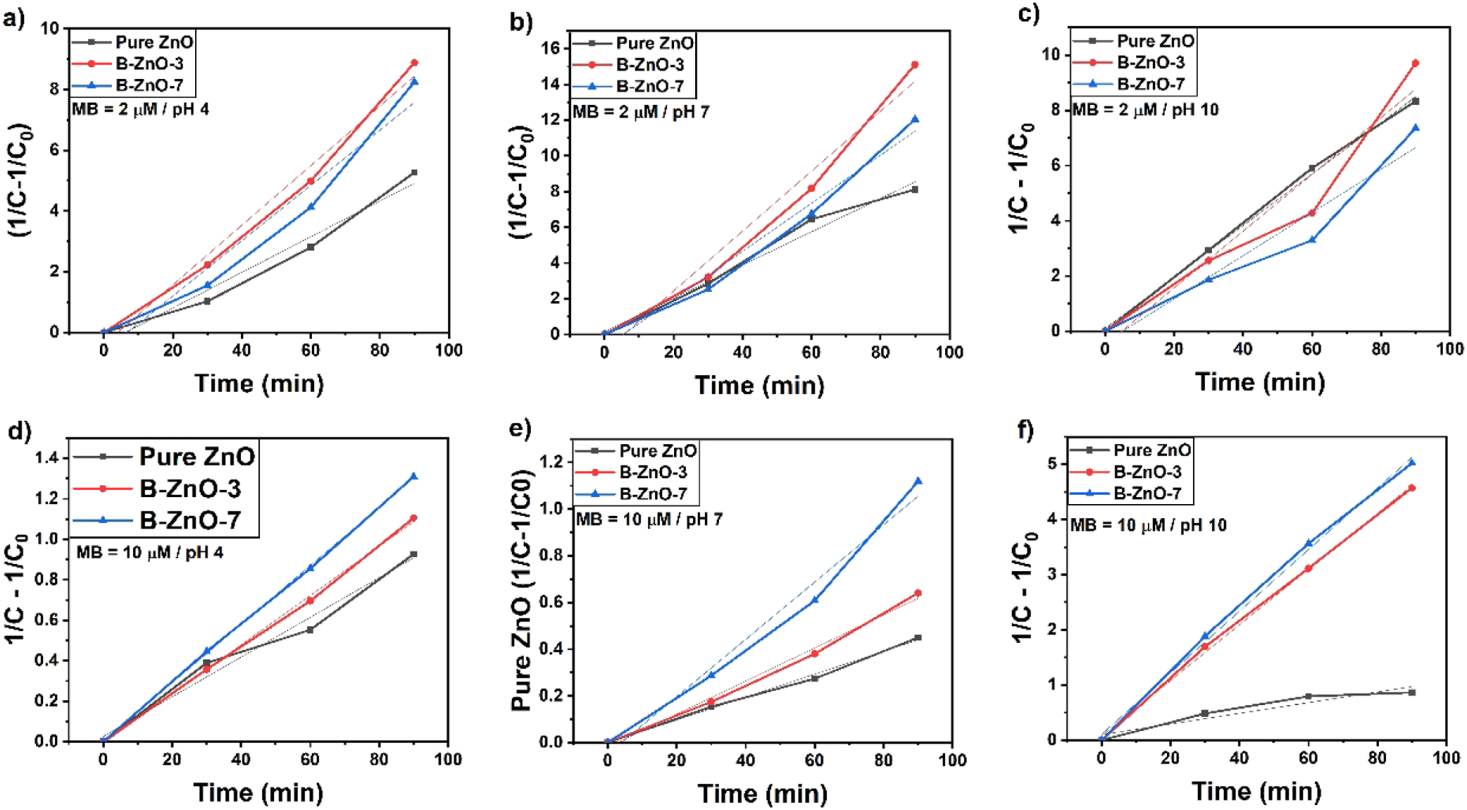
All samples pseudo-second order kinetics: a) MB, 2 μM and pH 4, b) MB, 2 μM and pH 7, c) MB, 2 μM and pH 10, d) MB, 10 μM and pH 4, e) MB, 10 μM and pH 7, f) MB, 10 μM and pH 10.

**Table 6:** Pseudo-Second Order Kinetic Rates and R^2^

Sample Name	MB Concentration (μM)	pH	Kinetic Rates (k_2_)	R^2^
ZnO	2	4	0.058617	0.9677
		7	0.093213	0.9822
		10	0.093225	0.9979
B-ZnO-3	2	4	0.098037	0.9838
		7	0.167730	0.9732
		10	0.102849	0.9387
B-ZnO-7	2	4	0.091017	0.9572
		7	0.134558	0.9768
		10	0.078296	0.9426
ZnO	10	4	0.009810	0.9783
		7	0.004902	0.9949
		10	0.009668	0.9064
B-ZnO-3	10	4	0.012175	0.9983
		7	0.007097	0.9916
		10	0.050421	0.9984
B-ZnO-7	10	4	0.014459	0.9996
		7	0.012263	0.9801
		10	0.055763	0.9967

The proposed photocatalytic degradation mechanism of MB using B-ZnO under UV-A light irradiation was illustrated in Figure 14. When ZnO absorbs photons with energy equal to or greater than its band gap, electrons transition from the valence band to the conduction band, generating e^–^-h^+^ pairs (eq 13). These charge carriers migrated to the surface and participated in redox reactions. To prevent recombination, e^–^-h^+^ scavengers such as oxygen and hydroperoxyl radicals play an important role. Without an electron scavenger, charge recombination occurred, resulting in energy dissipation as heat. In the conduction band, electrons interacted with oxygen to produce •O_2_^–^ anions. Then, this reaction continued until hydrogen peroxide was formed and •OH was formed by reacting with •O_2_^–^ (eqs 14–17). Hydrogen peroxide could also react with electrons to form •OH (eqs 18–19). Meanwhile, h^+^ in the valence band interacted with water and OH^–^ to form •OH (eqs 20–21). The •OHs produced enable the elimination of pollutants to harmless compounds such as water and carbon dioxide because •OH acts as a strong oxidizing agent (eq 22). The detailed reaction steps are as follows:

13 $$\mathrm{B:ZnO}+h\nu \to e^{-}+h^{+}$$

14 $$O_{2}+e^{-}\to •O_{2}^{-}$$

15 $$•O_{2}^{-}+H^{+}\to •HO_{2}$$

16 $$•HO_{2}+•HO_{2}\to H_{2}O_{2}+O_{2}$$

17 $$H_{2}O_{2}+•O_{2}^{-}\to •OH+OH^{-}+O_{2}$$

18 $$H_{2}O_{2}+h\nu \to 2•OH$$

19 $$H_{2}O_{2}+e^{-}\to •OH+OH^{-}$$

20 $$H_{2}O_{2}+h^{+}\to •OH+H^{+}$$

21 $$OH^{-}+h^{+}\to •OH$$

22 $$•OH+MB\to \mathrm{intermediates}\to CO_{2}+H_{2}O$$

**Figure 14:**
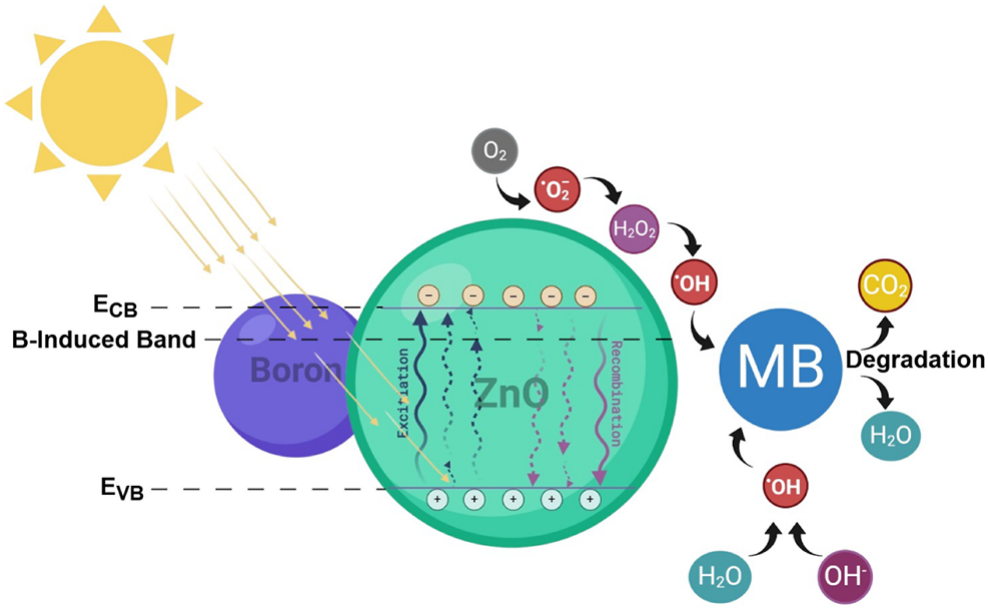
Illustration of B-ZnO photocatalytic degradation mechanism.

Boron doping enhanced the photocatalytic activity through several mechanisms reported in the literature. Atay et al. demonstrated that boron doping increased surface defects, as confirmed by photoluminescence studies.^53^ Boron doping also facilitated charge transport, acting as an electron carrier for reduction–oxidation reactions.^87^ Wang et al. observed that B-ZnO exhibited a 3.4 times higher photocurrent density compared to pure ZnO, indicating improved charge separation and reduced recombination rates.^52^ Similarly, Nguyen et al. suggested that boron, due to its electron-deficient nature, acted as an electron trap, preventing recombination and enhancing photocatalytic efficiency.^50^ Furthermore, boron-doped ZnO significantly reduced energy consumption for water treatment, making it an economically viable option for industrial applications. They calculated that the amount of electrical energy per unit in boron-doped material is five times lower than in pure ZnO.^50^

Electrical analyses confirmed that B-ZnO exhibited superior electrical conductivity in both DC and AC conditions, further enhancing its photocatalytic efficiency. Boron doping also induced a red shift in the UV–vis spectrum, reducing the band gap and improving conductivity. The decrease in the band gap directly increased the photocatalytic efficiency. This decrease indicated that new levels were formed there, and the electron conduction function increases. It has been suggested that boron on the ZnO surface attracts electrons and caused electrons in the valence band to move from the conduction band to boron before recombination. In this case, the boron atoms exhibited a behavior close to the conduction band and an n-type behavior.^88^ In this way, more electrons entered the system. New levels formed between the conduction and valence bands increased the electron-holding capacity by creating electron traps, thus reducing the recombination. XRD analyses indicated that boron doping introduced structural distortions, creating surface defects that serve as active sites for photocatalysis. The increased surface area of B-ZnO, as confirmed by SEM images, provided more active sites for dye adsorption, further promoting degradation. MB is a molecule with conjugated aromatic systems, that is, it contained stable π-π interactions.^89^ In high-pH environments, increased OH^–^ concentrations lead to the formation of more •OH, which efficiently break MB’s conjugated π-electron system, ultimately degrading it into CO_2_, H_2_O and small organic acids.^90^ Radical scavenging tests have shown that •OH is not the only dominant mechanism. Boron doping creates defect states and oxygen vacancies that act as electron-trapping centers. This suppresses charge recombination and facilitates the formation of •O_2_^–^. While this leads to an electron-driven photocatalytic pathway, the protected photogenerated holes (h^+^) also contribute significantly to the oxidation process. In contrast, the formation of •OH is less favored due to its indirect formation pathway. In summary, boron doping significantly enhanced ZnO’s photocatalytic performance by improving charge separation, increasing active surface sites, and promoting stronger adsorption of dye molecules. These combined effected contribute to the superior degradation efficiency of B-ZnO under UV-A irradiation.

Table 7 presented a comparative overview of the results obtained from the photocatalytic activity of boron-doped ZnO nanostructures against different pollutants, as reported in the literature. Compared to other studies, powder catalysts have generally been tested, and effective results have been obtained. However, very few studies have tested film structures. Additionally, as seen among the obtained thin-film photocatalysts, effective results are obtained over long periods; however, the photocatalyst obtained has been the most effective against MB in a short time, such as 90 min. Finally, the degradation of MB was evaluated under visible light (λ > 400 nm) using the B-ZnO-7 photocatalyst under constant experimental conditions. As illustrated in Figure 15, an impressive degradation efficiency of up to 93% was attained under visible light. This superior photocatalytic performance can be attributed to the narrowing of the bandgap due to boron doping, increased visible light absorption, and the effective suppression of the recombination of photogenerated electron–hole pairs.

**Figure 15:**
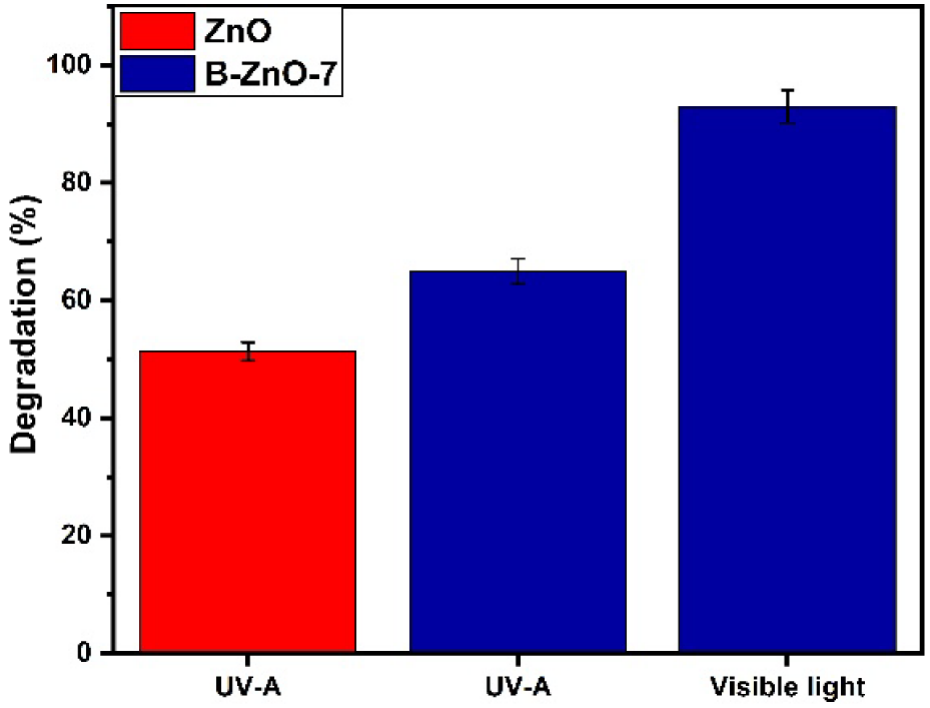
Comparison of MB degradation under UV-A and visible light.

**Table 7:** Comparison of B-ZnO Nanostructures to Photocatalytic Degradation^a^

Doped (w/w %)	Main Material	Time (min)	Light Source	Pollution	Degredation (%)	Type	Ref
1,5	ZnO	120	Solar Light	CN	89	Powder	^68^
6	ZnO	300	UV-A	MB	98	Film	^53^
15	ZnO	120	UV-A	RY 15	41	Film	^52^
2	ZnO	180	Visible Light	MB	54	Powder	^91^
5	ZnO	180	Solar Light	MG	90	Powder	^92^
5	ZnO	15	Solar Light	MB	70	Powder	^82^
4	ZnO	150	Solar Light	RB	90	Powder	^93^
-	ZnO	100	UV-A	RY 15	73	Film	^49^
3	ZnO	120	Hg Lamp	TCH	98	Powder	^50^
3	ZnO/TiO2	60	Hg Lamp	TCH	97	Powder	^57^
4	ZnO	60	Solar light	CIP	92	Powder	^56^
4	ZnO	80	UV–C	Congo red	98	Powder	^56^
3	ZnO	90	Hg Lamp	TCH	90	Powder	^54^
3	ZnO	90	UV-A	MB	62	Film	This work
7	ZnO	90	Visible Light	MB	93	Film	This work

a TCH: tetracycline hydrochloride, CIP: ciprofloxacin, CN: cyanide, RY 15: reactive yellow 15, MG: malachite green, RB: rhodamine B, MB.

### 3.6 Taguchi Design Analysis

Taguchi design was an optimization method used to obtain the best results at the lowest cost. In this study, the effects of various factors on the degradation efficiency and their individual contributions were analyzed and interpreted using the Taguchi method. It should be noted that the Taguchi orthogonal array design primarily evaluates the main effects of the parameters. Owing to the limited number of experimental runs, potential interaction effects between variables (e.g., reaction time × pH, doping level × initial concentration) were not explicitly analyzed, which is a recognized limitation of the Taguchi approach. Nevertheless, it offered a significant advantage by reducing the total number of experiments required, enabling efficient early stage optimization. Future studies might employ full factorial or response surface designs to investigate such interactions in greater detail. Characterization analyses and photocatalytic tests demonstrated that boron doping enhanced the photocatalytic activity of ZnO NRs. Our objective is to develop the most efficient photocatalysts under minimal conditions and investigate the impact of different factors on degradation performance. The degradation efficiencies (%) obtained from the Taguchi design experiments are given in Table 8.

**Table 8:** Experimental Design of Taguchi Method, Degradation Efficiency (%)

pH	Time (min)	MB Concentration (μM)	Doped (wt %)	Degradation (%)	S/N
4	30	2	0	11.3	21.0616
7	60	2	0	30.9	29.7992
10	90	2	0	35.8	31.0777
4	30	2	3	17.0	24.6090
7	60	2	3	39.2	31.8657
10	90	2	3	61.2	35.7350
7	30	2	7	17.6	24.9103
10	60	2	7	34.8	30.8316
4	90	2	7	42.2	32.5062
10	30	10	0	11.3	21.0616
4	60	10	0	17.3	24.7609
7	90	10	0	24.9	27.9240
7	30	10	3	11.3	21.0616
10	60	10	3	35.3	30.9555
4	90	10	3	29.8	29.4843
10	30	10	7	26.7	28.5302
4	60	10	7	24.7	27.8539
7	90	10	7	36.0	31.1261

In Taguchi analysis, the experimental results were evaluated using the “larger is better” criterion. The S/N performance analysis of the factors was conducted according to the Taguchi design method. Based on the analysis, the ranking of the most influential factors was as follows: pH, reaction time, initial pollutant concentration, and boron doping (%).

The order of the values and their effect ratios are shown in Table 9, while the main effect plot for the S/N ratio is illustrated in Figure 16. The rank values indicated the importance order of the factors, where a higher Δ*v*alue corresponded to a greater impact. According to the analysis, time was the most critical parameter, followed by the doping level, while pH also played a notable role. In contrast, concentration had the least effect on degradation efficiency. This ranking helps identify the key parameters for optimization, emphasizing that adjusting reaction time and doping levels was essential for enhancing photocatalytic performance.

**Figure 16:**
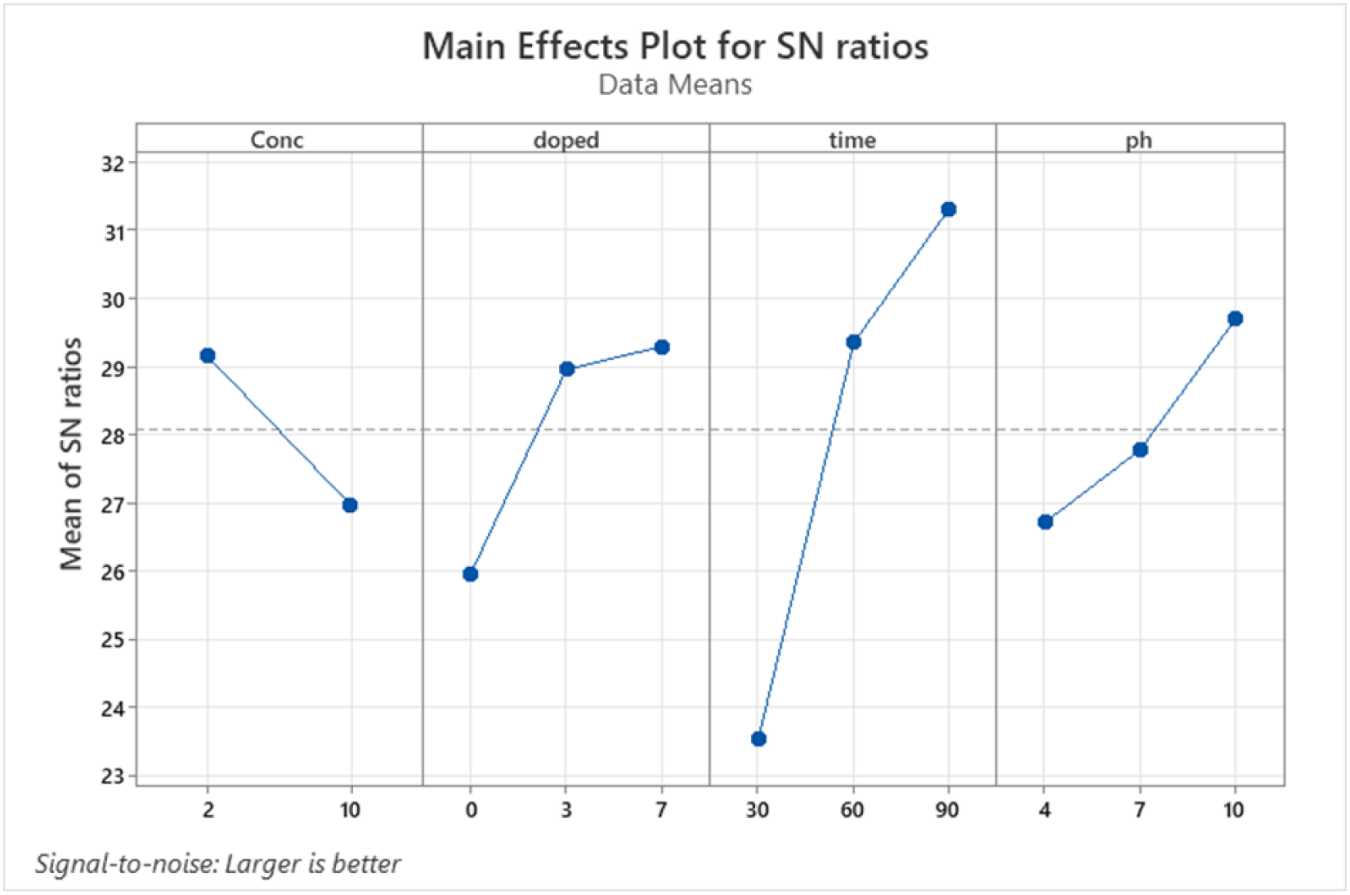
Main effect plot for the S/N ratio based on the “larger is better” criterion.

**Table 9:** Impact of Factors and Their Ranking

Level	pH	Time	MB Concentration (μM)	Doped (wt %)
1	26.71	23.54	29.19	25.95
2	27.78	29.34	26.97	28.95
3	29.30	31.31		29.29
Delta	2.99	7.77	2.18	3.35
Rank	3	1	4	2

The effect of pH could be explained by the electrostatic interaction between the catalyst surface and the target pollutant. pH affects both the surface state of the catalyst and the ionization state of the ionization state of organic compounds, thereby influencing the interfacial electron transfer and the photoredox process. Additionally, pH-dependent •OH production played a crucial role in reaction mechanisms that contribute to pollutant degradation, such as direct oxidation via positive holes and direct reduction via conduction band electrons. Since high pH levels enhanced •OH generation, higher degradation efficiency was expected at elevated pH values for MB photodegradation. According to the analysis, pH 10 was identified as the optimum condition.

Time was another critical factor in photocatalytic reactions. As reaction time increased, the photocatalytic efficiency improved up to a certain point, after which it reached saturation and remains constant. In this study, the optimum reaction time was determined to be 90 min. Concentration also played a significant role. Adjusting the concentration influenced the number of contaminants adhering to the catalyst surface and affected light penetration. At high concentrations, excessive accumulation of pollutants on the photocatalyst surface could inhibit degradation by blocking light absorption, preventing the catalyst from remaining active. In this study, the best degradation results were achieved at a concentration of 2 μM. Finally, the effect of the doping level was analyzed. As discussed in the previous section, increasing the boron doping level enhanced surface defects, suppressed the recombination process, and promoted the formation of e^–^-h^+^ pairs, facilitating ^e^lectron transport. Due to its small ionic radius, boron doping also increased the surface area of ZnO. This design model confirmed the accuracy of our findings regarding boron doping. While the lowest photocatalytic activity was observed in pure ZnO, the highest degradation efficiency was achieved with B-ZnO-7.

ANOVA analysis was conducted to examine the confidence levels in degradation efficiency. In this study, pH, concentration, reaction time, and doping level were considered independent variables, while the percentage of degradation served as the dependent variable. Additionally, error rates were calculated to evaluate the reliability of the model. The results, summarized in Table 10, indicated that the factors with the highest contribution to degradation efficiency were time (54.23%), doping level (12.73%), pH (12.20%), and concentration (10.24%), while the model’s error rate was determined to be 10.6%. ANOVA results were considered statistically significant when the p-value is less than 0.05.^61^ In this study, time (0.00012), doping level (0.01934), pH (0.02170), and concentration (0.01109) became significant, as their p-values were below the 0.05 threshold, confirming their substantial influence on the degradation process.

**Table 10:** ANOVA Analysis of Degradation Efficiency

Source	DF	Seq SS	Contribution	Adj SS	Adj MS	F-Value	P-Value
MB Concentration (μM)	1	293.6	10.24%	293.6	293.63	9.66	0.01109
Doped (wt %)	2	365.0	12.73%	365.0	182.52	6.01	0.01934
Time (min)	2	1554.9	54.23%	1554.9	777.46	25.59	0.00012
pH	2	349.8	12.20%	349.8	174.91	5.76	0.02170
Error	10	303.9	10.60%	303.9	30.39		
Total	17	2867.2	100.00%				

The R^2^ value indicated how well the data fits the model, with higher values representing a better fit. In this study, the R^2^ value was calculated as 89.4%. The resulting regression equation for MB removal is given in eq 23:

23 Degradation efficiency=−4,12−1,010⁡Conc+1,111⁡doped+0,3742⁡time+1,744⁡pH

Figure 17a showed how degradation changes over time and with doped amount. As shown in Figure 17a, irradiation time was identified as an effective parameter that enhanced degradation for all dopant levels. This trend was expected and confirmed that the photocatalyst functioned effectively under UV irradiation. A quasi-linear increase in photodegradation with time was observed up to a certain duration, which is characteristic of photocatalytic systems operating under kinetically controlled conditions. Among the studied samples, the highest degradation efficiency was obtained for the B-ZnO-3 sample. Figure 17b presented how the degradation rate varies with the doped amount and pH. For pure ZnO, photodegradation was more efficient in the neutral pH region. This behavior was attributed to the positively charged ZnO surface under acidic conditions, which limited dye adsorption due to electrostatic repulsion, as MB is a cationic dye. In the basic region, the increased concentration of OH^–^ were likely to enhance charge carrier recombination, resulting in reduced photocatalytic efficiency. In contrast, the introduction of B-ZnO-3 suppressed e^—^h^+^ recombination under basic conditions, thereby facilitating greater •OH formation and improving degradation performance. A lower degradation efficiency was observed for the B-ZnO-7 sample compared to the B-ZnO-3 sample, which was attributed to an increased density of surface defects acting as recombination centers at higher dopant concentrations.

**Figure 17:**
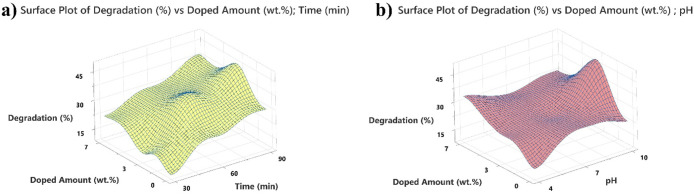
Response surface plots showing the effect of doped amount (wt.%) on photocatalytic degradation efficiency (%) as a function of a) reaction time (min) and b) solution pH.

Figure 18 showed the normal probability plot, which assesses the model’s reliability. The closer the data points are to the reference line, the better the model’s fit. In this study, the normal probability plot indicated that the model aligned well with the experimental data, confirming its validity.

**Figure 18:**
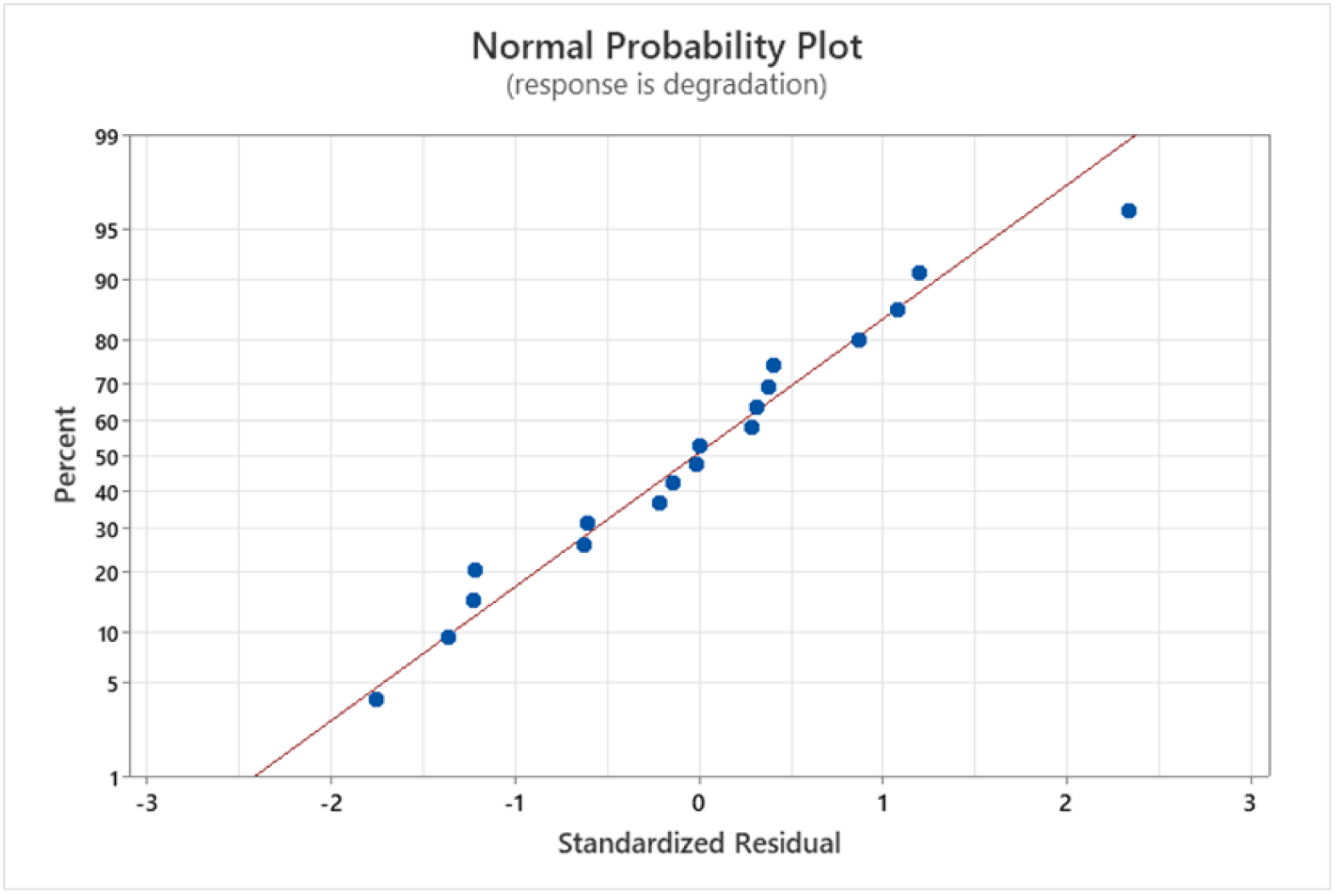
Normal probability plot for the degradation efficiency model.

According to Figure 16, the highest degradation efficiency was achieved at pH 10, 90 min of reaction time, B-ZnO-7, and an MB concentration of 2 μM. The optimal conditions determined through optimization aligned well with the characterization analyses of B-ZnO and the proposed possible photocatalytic mechanisms.

## 4 Conclusion

This study aimed to enhance the photocatalytic and electrical properties of ZnO NRs, a metal oxide semiconductor, through boron doping via the hydrothermal method. The optimum conditions for the synthesized photocatalyst were determined using Taguchi method, minimizing the number of experiments while evaluating the effects of pH, reaction time, concentration, and doping level. Experimental results confirmed the successful synthesis of pure and B-ZnO NRs (3 wt.%, 7 wt.%). The samples were characterized using XRD, FT-IR, SEM, AFM, and UV–vis analyses. XRD patterns revealed that the ZnO NRs predominantly grew in the (002) orientation, with increased (100) orientation upon boron doping. Due to the small atomic radius of boron, a peak shift toward lower angles was observed, confirming its successful incorporation into the ZnO crystal structure. FT-IR analysis indicated the presence of B–O and B–O–B stretching vibrations within the 1300–1700 cm^–1^ range, further verifying boron doping. SEM and AFM images showed morphological distortions and changes in crystal size due to doping. UV–vis spectroscopy results demonstrated enhanced absorption in the visible light region for boron-doped samples, indicating a reduced band gap. Calculations revealed a band gap reduction from 3.22 eV (pure ZnO) to 2.95 eV for B-ZnO-7. Temperature-dependent AC-DC electrical analyses revealed that conductivity increased with boron doping, with a transition from semiconducting to metallic behavior. This phenomenon is attributed to band gap narrowing and electron donation by boron, enhancing charge carrier mobility. Boron doping introduced electron traps, reducing recombination rates and improving charge separation.

Photocatalytic tests confirmed that boron-doped ZnO exhibited superior degradation performance compared to pure ZnO. At pH 10 and 2 μM MB concentration, B-ZnO-3 showed a 70% increase in photocatalytic efficiency, while B-ZnO-7 demonstrated a 140% increase at pH 10 and 10 μM MB concentration. The enhanced photocatalytic activity is attributed to improved charge transport, a reduced recombination rate due to surface defects, increased surface area, and enhanced photostability. The Taguchi method identified the optimum conditions as pH 10, 2 μM MB concentration, 90 min of reaction time, with B-ZnO-7 as the most effective catalyst. Statistical validation using confirmed the significance of the model, with an R^2^ value of 89%, indicating a strong fit.

Overall, this study demonstrated that boron doping significantly enhanced both photocatalytic efficiency and electrical conductivity—improving DC conductivity by 5-fold and AC conductivity by 3-fold. The results highlight the potential B-ZnO NRs for photocatalytic applications, pollutant degradation, future advancements in hydrogen storage and sensor technologies, and energy storage systems.

Subsequent radical scavenger experiments revealed that the dominant active species in the photodegradation process were •O_2_^–^, h^+^ and •OH, in that order. Photocatalytic tests conducted under visible light yielded an approximate removal efficiency of 93%, indicating highly promising performance of the material in the visible region. Additionally, five consecutive cycle tests were conducted to evaluate the photocatalyst’s mechanical stability and reusability. The results showed that the catalyst remained stable with minimal performance loss over five cycles and that the B-ZnO nanorods largely retained their high photocatalytic activity and structural stability with repeated use.

Although the results are promising, this study has certain limitations. The photocatalytic tests were limited to MB, and the visible light tests need to be expanded. Future studies will address the practical applicability of B-ZnO NRs and evaluate them in greater detail.

## Supplementary Material



## Data Availability

Data will be made available upon request.

## Notes

SEM images to pure ZnO (Figure S1); SEM images to B-ZnO-3 (Figure S2); SEM images of B-ZnO-7 (Figure S3); I–V characteristics for pure ZnO NRs, b) for B-ZnO-3, c) for B-ZnO-7 (Figure S4); UV–Vis spectrum of adsorption under dark conditions at 40 min a) pure ZnO, b) B-ZnO-3 (Figure S5); UV–vis spectrum at pH 10 for MB-2: a) pure ZnO, b) B-ZnO-3 (Figure S6); B-KA lines spectrum of a) pure glass, b) ZnO NRs, c)­B-ZnO-3 NRs, d) B-ZnO-7 NRs (Figure S7); pseudo-second order kinetic rates and R^2^ (Table S1); boron content of B-ZnO-3 and B-ZnO-7 determined by WD-XRF analysis (Table S2) (PDF)
