# Supplementary material for: Enhanced Photocatalytic and Electrical Performance of Boron-Doped ZnO Nanorods: A Taguchi Optimization Approach for Degradation Efficiency
Source: ACS Omega. 2026 May 25;11(22):32002–22. doi: 10.1021/acsomega.5c11437 (PMC13261602; doi:10.1021/acsomega.5c11437)
Supplement: Supplementary file 1 [file ao5c11437_si_001.pdf]

Enhanced Photocatalytic and Electrical Performance of Boron-Doped ZnO Nanorods: A Taguchi Optimization Approach for Degradation Efficiency

Eray TABAK[a,b,c*], Sadullah ÖZTÜRK[b,c], Arif KÖSEMEN[b,c], Sahika Sena BAYAZİT[b,c], Necmettin KILINC[d], Mika Sillanpää[e,f,g], Birgül BENLİ[a,h*]

[a] *Istanbul Technical University, Graduate School, Nano Science and Nano Engineering Programme, 34469, Istanbul, Türkiye*
[b] *Istanbul University-Cerrahpasa, Institute of Nanotechnology and Biotechnology, 34500, Istanbul, Türkiye*
[c] *Health Biotechnology Joint Research and Applications Center of Excellence, 34098, Istanbul, Türkiye*
[d] *Inonu University, Faculty of Science & Arts, Department of Physics, 44280, Malatya, Türkiye*
[e] *Saveetha School of Engineering, Saveetha Institute of Medical and Technical Sciences, Saveetha University, Chennai, Tamil Nadu – 602105, India*
[f] *Institute for Nanotechnology and Water Sustainability (iNanoWS), Florida Campus, College of Science, Engineering and Technology, University of South Africa, Johannesburg, 1709, South Africa*
[g] *Centre of Research Impact and Outcome, Chitkara University Institute of Engineering and Technology, Chitkara University, Rajpura-140401, Punjab, India*
[h] *Istanbul Technical University, Faculty of Mines, Department of Mineral Processing Engineering, 34469, Istanbul, Türkiye*

**E-Mail: eray.tabak@iuc.edu.tr; benli@itu.edu.tr*

Supplementary Figure Captions:

Figure S1: SEM images to pure ZnO

Figure S2: SEM images to B-ZnO-3

Figure S3: SEM images to B-ZnO-7

Figure S4: I-V characteristic for pure ZnO NRs, b) for B-ZnO3, c) for B-ZnO-7

Figure S5: UV–Vis Spectrum of Adsorption under dark donditions at 40 min a) pure ZnO, b) B-ZnO-3.

Figure S6: UV-Vis spectrum at pH 10 for MB-2 a) Pure ZnO, b) B-ZnO-3.

Figure S7: B-KA lines spectrum of a) pure glass, b) ZnO NRs, c)B-ZnO-3 NRs, d) B-ZnO-7 NRs.

Supplementary Table Captions:

Table S1: Pseudo-second order kinetic rates and R^2

Table S2: Boron content of B-ZnO-3 and B-ZnO-7 determined by WD-XRF analysis.

Supplementary Figures:

Figure S1: SEM images to pure ZnO

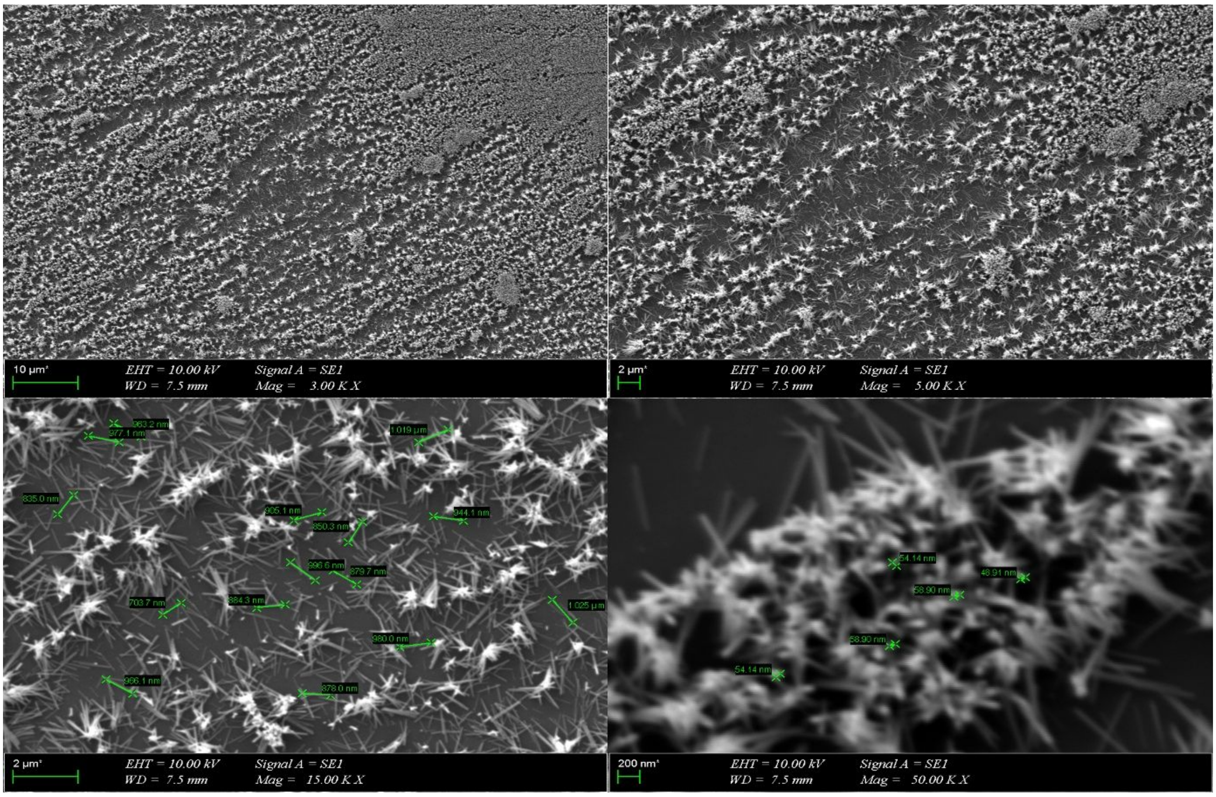

Figure S2: SEM images to B-ZnO-3

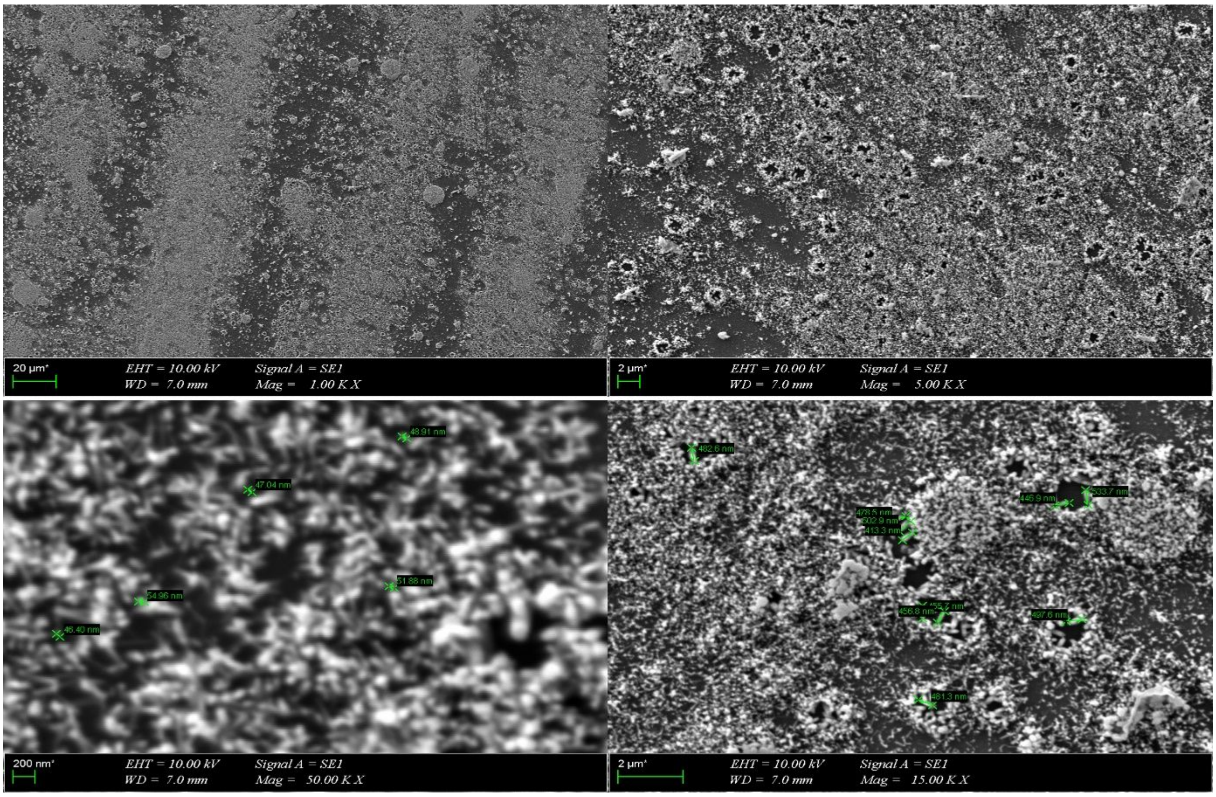

Figure S3: SEM images to B-ZnO-7.

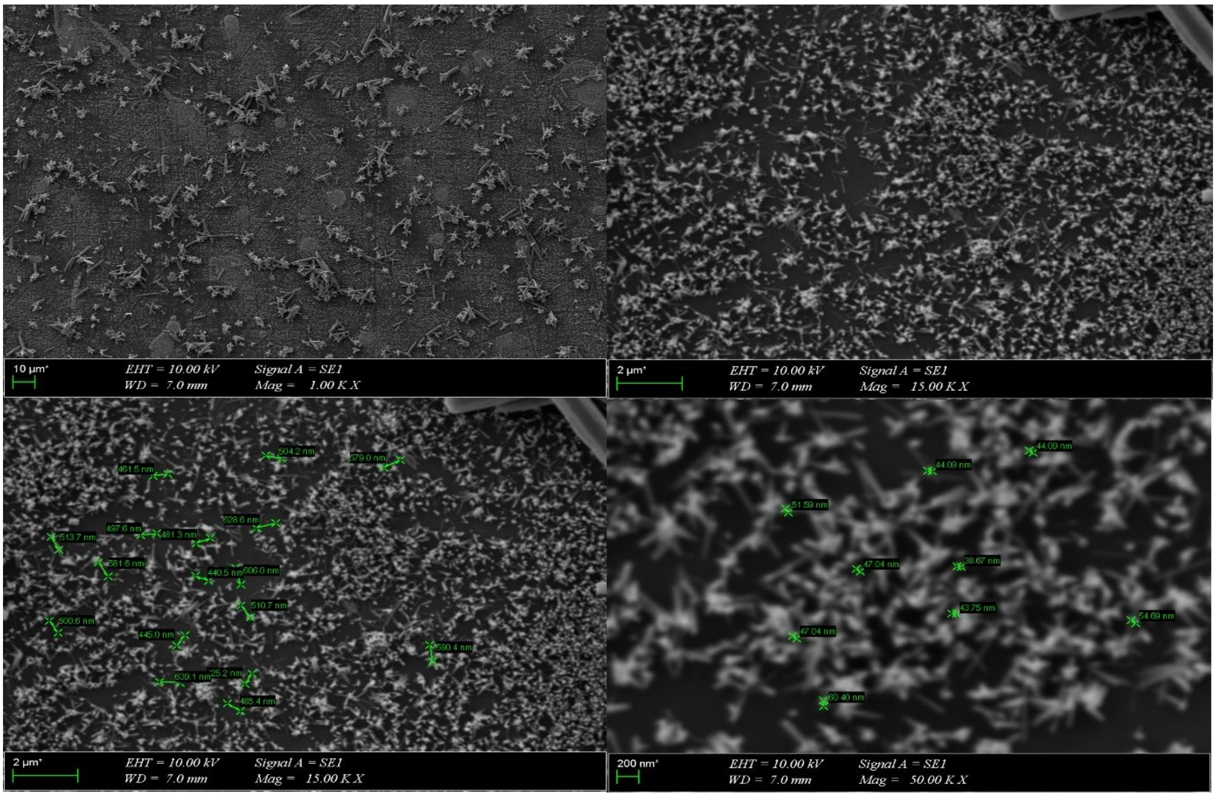

Figure S4: I-V characteristic for pure ZnO NRs, b) for B-ZnO3, c) for B-ZnO-7

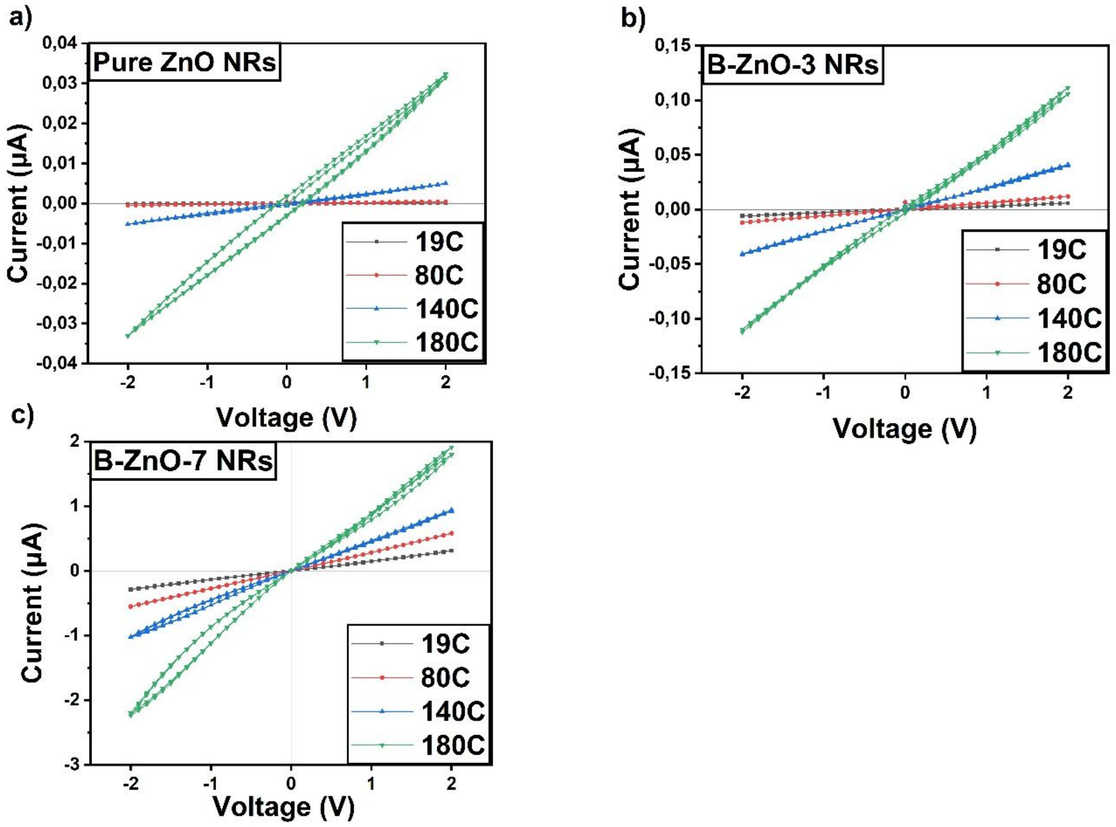

Figure S5: UV–Vis Spectrum of Adsorption under Dark Conditions at 40 min a) pure ZnO, b) B-ZnO-3.

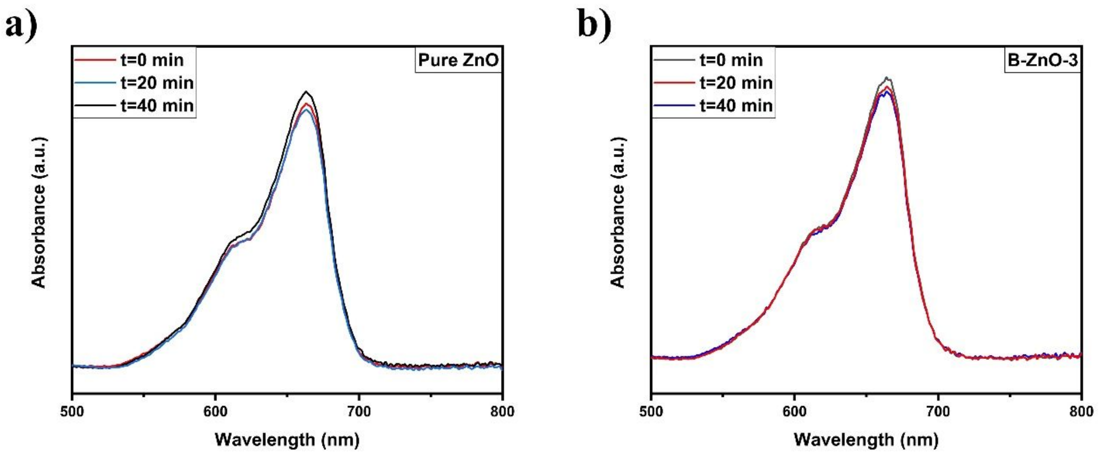

Figure S6: UV-Vis spectrum at pH 10 for MB-2 a) Pure ZnO, b) B-ZnO-3.

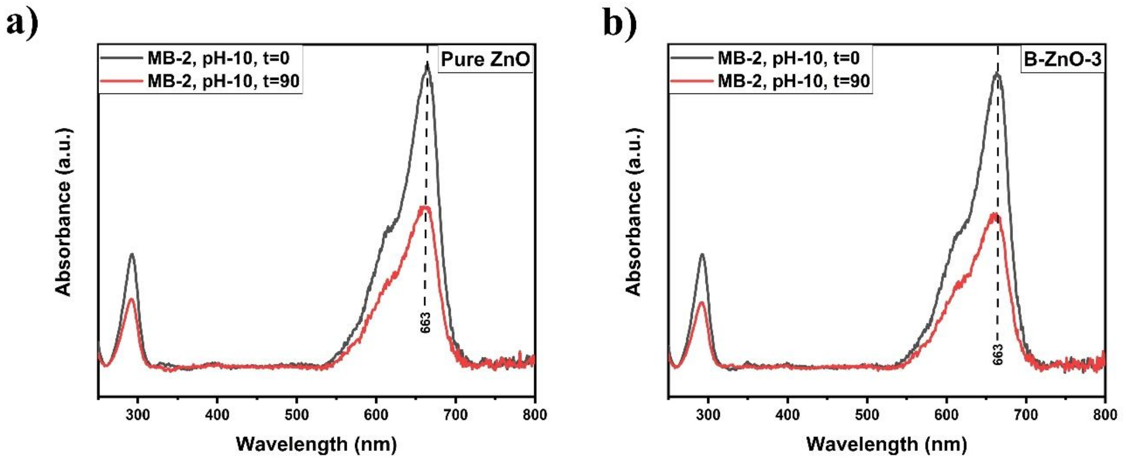

Figure S7: B-KA lines spectrum of a) pure glass, b) ZnO NRs, c)B-ZnO-3 NRs, d) B-ZnO-7 NRs.

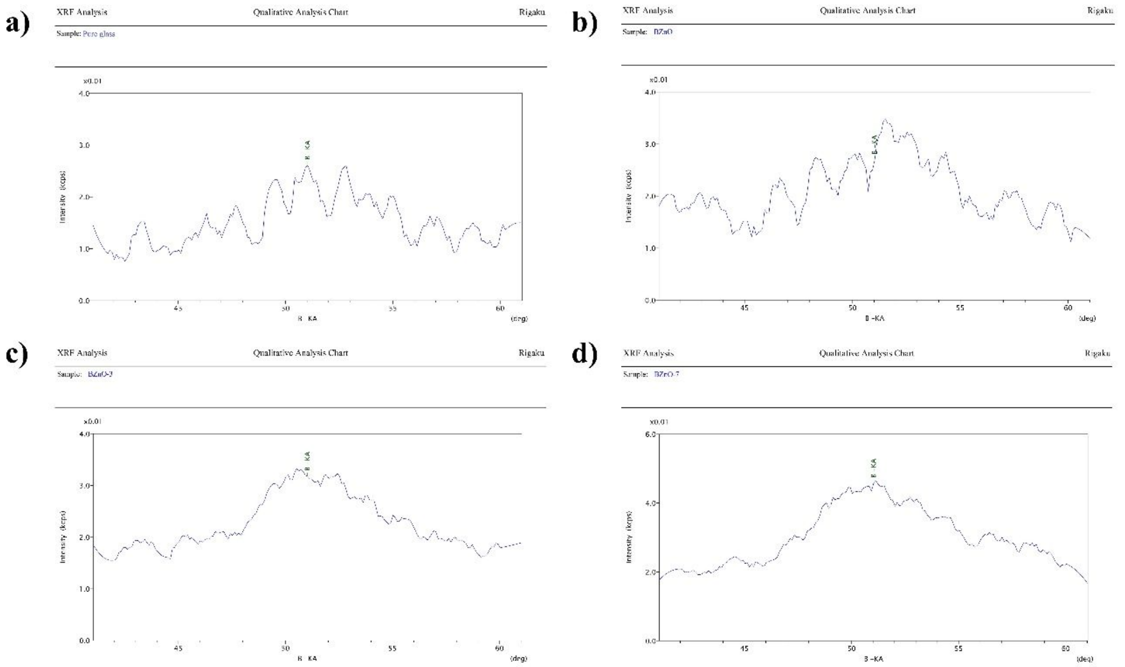

Supplementary Table:

Table-S1: Pseudo-second order kinetic rates and R^2

Sample Name	MB Concentration (μM)	pH	Kinetic Rates (k_2)	R^2
		4	0.058617	0.9677
ZnO	2	7	0.093213	0.9822
		10	0.093225	0.9979
		4	0.098037	0.9838
B-ZnO-3	2	7	0.167730	0.9732
		10	0.102849	0.9388
		4	0.091017	0.9572
B-ZnO-7	2	7	0.134558	0.9768
		10	0.078296	0.9426
		4	0.00981	0.9783
ZnO	10	7	0.004902	0.9949
		10	0.009668	0.9063
		4	0.012175	0.9983
B-ZnO-3	10	7	0.007097	0.9916
		10	0.050421	0.9984
		4	0.014459	0.9996
B-ZnO-7	10	7	0.012263	0.9801
		10	0.055763	0.9967

Table S2: Boron content of B-ZnO-3 and B-ZnO-7 determined by WD-XRF analysis.

Sample Name	B-ZnO-3 NRs	B-ZnO-7 NRs
**Boron amount (%)**	2,55	5,79
